# It takes two transducins to activate the cGMP-phosphodiesterase 6 in retinal rods

**DOI:** 10.1098/rsob.180075

**Published:** 2018-08-01

**Authors:** Bilal M. Qureshi, Elmar Behrmann, Johannes Schöneberg, Justus Loerke, Jörg Bürger, Thorsten Mielke, Jan Giesebrecht, Frank Noé, Trevor D. Lamb, Klaus Peter Hofmann, Christian M. T. Spahn, Martin Heck

**Affiliations:** 1 Institut für Medizinische Physik und Biophysik, Charité – Universitätsmedizin Berlin, corporate member of Freie Universität Berlin, Humboldt-Universität zu Berlin, and Berlin Institute of Health, Berlin, Germany; 2 Department of Mathematics, Computer Science and Bioinformatics, Freie Universität Berlin, Berlin, Germany; 3 Microscopy and Cryo Electron Microscopy Group, Max-Planck Institut für Molekulare Genetik, Berlin, Germany; 4 Eccles Institute of Neuroscience, John Curtin School of Medical Research, Australian National University, Canberra, Australian Capital Territory 2600, Australia; 5 Zentrum für Biophysik und Bioinformatik, Humboldt-Universität zu Berlin, Berlin, Germany

**Keywords:** PDE6, visual signal transduction, coincidence switch, density switch, noise filtering

## Abstract

Among cyclic nucleotide phosphodiesterases (PDEs), PDE6 is unique in serving as an effector enzyme in G protein-coupled signal transduction. In retinal rods and cones, PDE6 is membrane-bound and activated to hydrolyse its substrate, cGMP, by binding of two active G protein α-subunits (Gα*). To investigate the activation mechanism of mammalian rod PDE6, we have collected functional and structural data, and analysed them by reaction–diffusion simulations. Gα* titration of membrane-bound PDE6 reveals a strong functional asymmetry of the enzyme with respect to the affinity of Gα* for its two binding sites on membrane-bound PDE6 and the enzymatic activity of the intermediary 1 : 1 Gα* · PDE6 complex. Employing cGMP and its 8-bromo analogue as substrates, we find that Gα* · PDE6 forms with high affinity but has virtually no cGMP hydrolytic activity. To fully activate PDE6, it takes a second copy of Gα* which binds with lower affinity, forming Gα* · PDE6 · Gα*. Reaction–diffusion simulations show that the functional asymmetry of membrane-bound PDE6 constitutes a coincidence switch and explains the lack of G protein-related noise in visual signal transduction. The high local concentration of Gα* generated by a light-activated rhodopsin molecule efficiently activates PDE6, whereas the low density of spontaneously activated Gα* fails to activate the effector enzyme.

## 1. Introduction

Cyclic nucleotide phosphodiesterase enzymes (PDEs) play important regulatory roles in diverse signal transduction cascades by degrading the second messenger cyclic nucleotides cAMP and cGMP [1–3]. The PDE superfamily comprises 11 families with 21 genes in mammals [4]. Commonly, PDEs have a role in the recovery phase of signal transduction cascades [5], but phosphodiesterase 6 (PDE6) acts as the activating effector of visual signal transduction in retinal rods and cones.

Vision starts with the absorption of a photon by the photosensitive molecule rhodopsin. The active form of rhodopsin (R*) catalyses the exchange of bound GDP for GTP in many copies of the heterotrimeric G-protein transducin (G). The activated GTP-bound α-subunit of G (Gα*) binds and thereby activates PDE6. The rapid degradation of cGMP by active PDE6 causes the closure of cGMP-gated channels, membrane hyperpolarization and neuronal response [6,7]. Receptor, G-protein and effector are all associated with the membranes of flat disc vesicles (in rods) or evaginations (in cones) that are stacked in the outer segments of the photoreceptor cells [8]. Membrane binding of PDE6 is mediated by C-terminal isoprenylation [9].

All PDEs comprise N-terminal regulatory/targeting domains and conserved C-terminal catalytic domains. Most PDEs are homodimeric and are activated by interaction with partner proteins and/or cofactors through their N-terminal regulatory domains [2]. Five of the 11 PDE families feature cGMP-binding tandem GAF (c*G*MP-specific PDEs, *a*denylyl cyclases and *F*hlA) domains at their N-terminus and are directly activated by binding of cyclic nucleotides [10], as is well characterized for PDE2 and PDE5 [11]. Again, retinal rod PDE6 is an exception in that it is heterodimeric and features N-terminal tandem GAF domains that are likely to be permanently occupied by cGMP in mammalian rod cells [12]. Another difference from other PDEs is that the PDE6 holoenzyme (holo-PDE6) comprises two additional inhibitory PDE6γ subunits (approx. 10 kDa each). The PDE6γ subunits span the two PDE6 catalytic subunits (approx. 100 kDa each) from N- to C-terminus [13,14] and maintain inhibition of the holoenzyme by blocking access of cGMP to the C-terminal catalytic pockets [15,16]. Upon binding to membrane-associated holo-PDE6, Gα* displaces the C-termini of the PDE6γ subunits from the catalytic cGMP-binding sites, thereby releasing their inhibitory constraint on PDE6αβ [17,18].

While the catalytic domains of all PDEs, except PDE6, have been structurally characterized [4], no high-resolution description of a PDE holoenzyme is available. However, a crystal structure for a truncated PDE2A comprising GAFa, GAFb and the catalytic domain was solved [19]. For PDE6, several low-resolution negative-stain electron microscopy (EM) structures have been published [20–22]. A recent cryo-EM study confirmed the overall structural organization of PDE6 [23], but a conclusive model of the PDE6 activation mechanism is still lacking. It is well accepted that disc membrane-associated PDE6 is fully activated when two copies of Gα* are bound. However, the enzymatic activity of the membrane-bound intermediary 1 : 1 Gα* · PDE6 complex and the affinity of Gα* for the two binding sites on PDE6, and possible allosteric and/or cooperative effects, remain elusive. Here, we present the results of a combined enzymatic, computational and structural investigation of bovine rod PDE6. Our results reveal that the first Gα* interacts with membrane-bound PDE6 with high affinity, followed by the second Gα* binding with low affinity. The low level of cGMP hydrolytic activity with only a single Gα* bound establishes a functional asymmetry of mammalian rod PDE6 in the presence of membranes, which allows sequestering of spontaneously activated G proteins in a functionally inactive form. We thereby provide an explanation for the previously suggested difference in activity between spontaneously and light-activated PDE6 [24]. The identified properties of the PDE6 effector enzyme have the capacity to keep the noise of the rod cell low enough to allow the reliable detection of single quanta of light.

## 2. Material and methods

### 2.1. Protein and membrane preparations

Rod outer segments were prepared from frozen bovine retinas as described [25]. Isolated disc membranes were prepared from rod outer segments by two consecutive extractions with low salt buffer as described [26]. Rhodopsin concentration was determined from its absorption spectrum using *ɛ*_500_ = 40 000 M^−1^ cm^−1^.

Native G-protein (transducin) was extracted from bovine rod outer segments as described [27,28]. Gα and Gβγ subunits were separated on a Blue-Sepharose column (1 ml HiTrap Blue, GE Healthcare, Freiburg, Germany) as described [29] and concentrated to 20 µM (centricon YM10, Millipore, Schwalbach, Germany). GαGTPγS (Gα*) was prepared by activation of isolated Gα (20 µM) with twofold molar excess of GTPγS (10 min incubation at room temperature) in the presence of 0.5 µM rhodopsin in isolated disc membranes. After removal of the membranes by centrifugation, isolated Gα* was stored at −40°C. Native PDE6 was extracted and purified from bovine rod outer segments as described [28,30]. Briefly, PDE6 was purified by TSK-heparin column chromatography, dialyzed against 20 mM BTP (pH 7.5), 130 mM NaCl, 1 mM MgCl_2_ and 1 mM TCEP, and concentrated to 10–20 µM (Centricon YM30, Millipore). Purified PDE6 was stored at −40°C in 20% glycerol. Note that PDE6 preparations with detectable basal hydrolytic cGMP activity were discarded, because basal activity indicates proteolytic activation of PDE6 and reduced membrane binding (see below). For electron microscopy, PDE6 (50 µl) was further purified prior to the experiments by gel filtration (Äktamicro System, GE Healthcare, Freiburg, Germany) in 20 mM BTP (pH 7.5), 130 mM NaCl, 1 mM MgCl_2_ and 1 mM TCEP at 4°C using Superdex 200 GL 5/150 columns (GE Healthcare, Freiburg, Germany).

A truncated, active form of PDE6 (tPDE6) was generated by limited trypsination as described [9]. For preparation of tPDE6, purified PDE6 (4 mg) was incubated with bovine pancreas trypsin (0.4 mg; Sigma-Aldrich, Munich, Germany) on ice for 20 min. Subsequently, soya bean trypsin inhibitor (4 mg; Sigma-Aldrich) was added to stop the reaction and the resulting mixture was immediately subjected to gel filtration in 20 mM BTP (pH 7.5), 130 mM NaCl, 1 mM MgCl_2_ and 1 mM TCEP (Superdex 200, GE Healthcare, Freiburg, Germany). The resulting pure tPDE6 was concentrated to 10–20 µM (Centricon YM30, Millipore) and stored with 20% glycerol at −40°C. Proteolytic removal of PDE6γ was confirmed by measuring enzymatic tPDE6 activity and by SDS–PAGE. Prior to the EM experiments, tPDE6 was again purified by gel filtration (Äktamicro System, GE Healthcare) in 20 mM BTP (pH 7.5), 130 mM NaCl, 1 mM MgCl_2_ and 1 mM TCEP at 4°C using Superdex 200 GL 5/150 columns (GE Healthcare).

### 2.2. Quantification of phosphodiesterase 6 membrane association

A centrifugal pull-down assay was carried out to separate and quantify membrane association of PDE6 in parallel with the activity measurements. Samples (50 µl) containing PDE6, Gα*, isolated disc membranes and 2.5 mM cGMP were subjected to centrifugation at 14 000*g* for 5 min at 22°C. After removing the supernatant, the pellet was washed once with 50 µl of buffer. Supernatant and pellet fractions were subjected to SDS–PAGE analysis. PDE6 was densitometrically quantified in Coomassie-stained gels (GelAnalyzer). The results described below show that, on average, 66 ± 3% of PDE6 is membrane-bound in the Gα* titration experiments.

### 2.3. Phosphodiesterase 6 activity measurements

PDE6 catalysed hydrolysis of cGMP (or 8-Br-cGMP) generates GMP (or 8-Br-GMP) and a proton. The rate of hydrolysis was monitored in real time using a fast-response micro-pH electrode (Radiometer PHC3359-8, Hach Lange GmbH, Düsseldorf, Germany) as described [31]. All measurements were performed in 120 µl final volume at 22°C in buffer (pH 7.5) containing 130 mM NaCl, 1 mM MgCl_2_, 1 mM TCEP and 4 mM BTP (for 8-Br-cGMP) or 20 mM BTP (for cGMP). PDE6, Gα* and disc membranes were added as indicated in the figure legends. All samples were incubated with 50 µM cGMP for 10 min prior to the measurements to saturate the non-catalytic cGMP-binding sites of PDE6. Reactions were then initiated by the addition of 2.5 mM cGMP (or 8-Br-cGMP) and the change in pH of the sample was monitored over time (50–200 ms dwell time). Note that the nucleotide concentration used is well above the *K*_m_ values of PDE6 for cGMP (15 µM [4]) or 8-Br-cGMP (160 µM [32]), respectively. PDE6 activity was estimated from the slope of the initial, linear range of the resulting pH change. After complete cGMP hydrolysis, samples were titrated with 0.1 mol l^−1^ NaOH in order to relate the measured pH changes to the concentration of cGMP hydrolysed.

### 2.4. Analysis of titration curves

**Figure 1.**
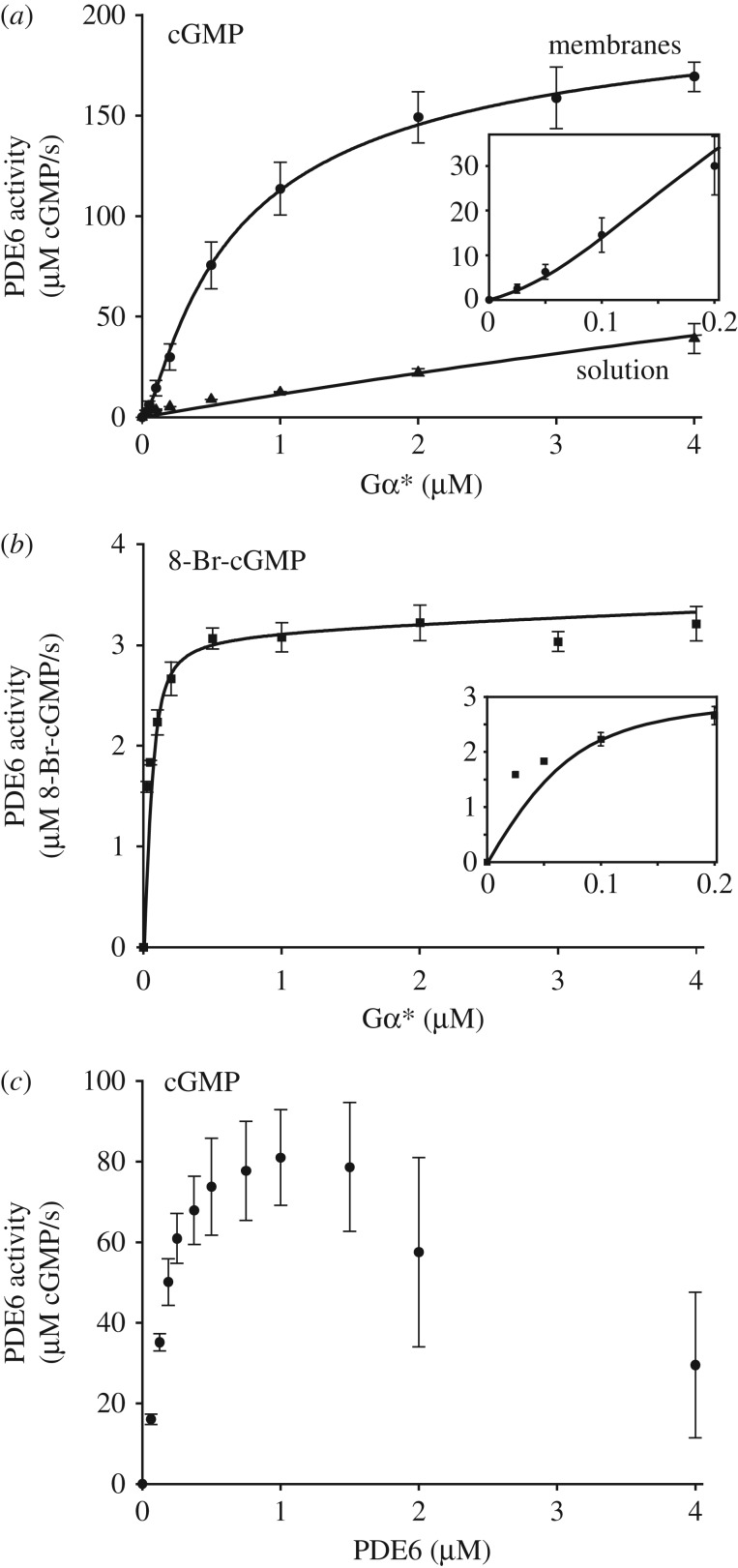
Activity of PDE6 stimulated by GTPγS-activated G protein α-subunit (Gα*). (*a*) Activity of PDE6 (0.1 µM) measured as the rate of cGMP hydrolysis at increasing Gα* concentrations in solution (triangles) or in the presence of disc membranes (circles; 10 µM rhodopsin). Note that for PDE6 in solution, the data are limited to the initial part of the binding curve. (*b*) PDE6 (0.1 µM) activity of same batches of samples measured in the presence of disc membranes (10 µM rhodopsin) with 8-Br-cGMP. Insets in (*a*) and (*b*) depict the lower concentration range measured in the presence of membranes on expanded scales. Note that no basal PDE6 activity (0 µM Gα*) was detectable in (*a*) and (*b*). Note also the different *y*-scales. (*c*) PDE6 activity as a function of PDE6 concentration at fixed Gα* (0.25 µM) in the presence of disc membranes (10 µM rhodopsin). All data points represent the average of three experiments with error bars depicting standard errors. Solid lines represent best fits to the data points (see the text for details). Note that the relatively large error bars in (*a*) and (*c*) are due to averaging measurements with different preparations (Gα*, PDE6 and membranes) and different combinations of those preparations. The data points for individual titration curves show little scatter (see electronic supplementary material, figure S2) and the main characteristics of the curves (sigmoidal shape in (*a*) and biphasic curve in (*c*)) were well established in each titration experiment.

The rate of PDE6 catalysed cGMP hydrolysis (*v*) resulting from titration of soluble PDE6 (PDE6_s_) with Gα* (figure 1*a*; electronic supplementary material, figure S1*a*) was fitted with a Michaelis–Menten kinetics hyperbolic function:2.1
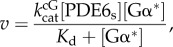
where [PDE6_s_] represents the overall concentration of individual catalytic subunits of PDE6_s_ and *K*_d_ the apparent dissociation constant of the Gα*/PDE6 complex in solution, reflecting the finding that the apparent affinity of Gα* for the two Gα* binding sites on soluble PDE6 is identical. In the fit, the maximum reaction rate 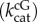 of soluble PDE6 was fixed to the value obtained for membrane-associated PDE6 (see below and electronic supplementary material, table S2).

**Scheme 1.**
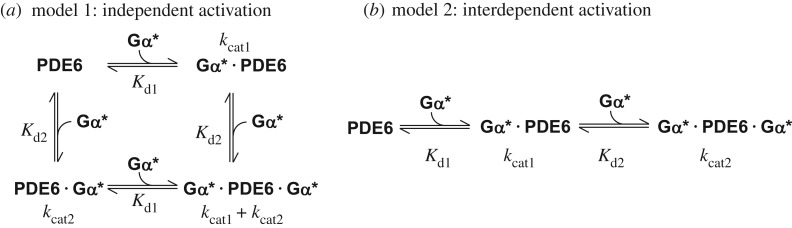
Reaction models of PDE6 activation. In model 1 (*a*, independent activation), membrane-bound PDE6 is assumed to comprise two independent but non-identical Gα*-binding sites (see the text and table 1 for details). In model 2 (*b*, interdependent activation), the two Gα*-binding sites on membrane-bound PDE6 are initially identical. Binding of Gα* to either of these two sites induces a conformational change in the PDE6 that results in altered affinity for the second Gα*. Note that in all our enzymatic measurements, PDE6 was incubated with a permanently activated Gα-subunit (GTPγS-activated Gα; Gα*), i.e. the two equilibrium models are well suited for analysis of the titration curves depicted in figure 1*a*,*b*.

**Figure 2.**
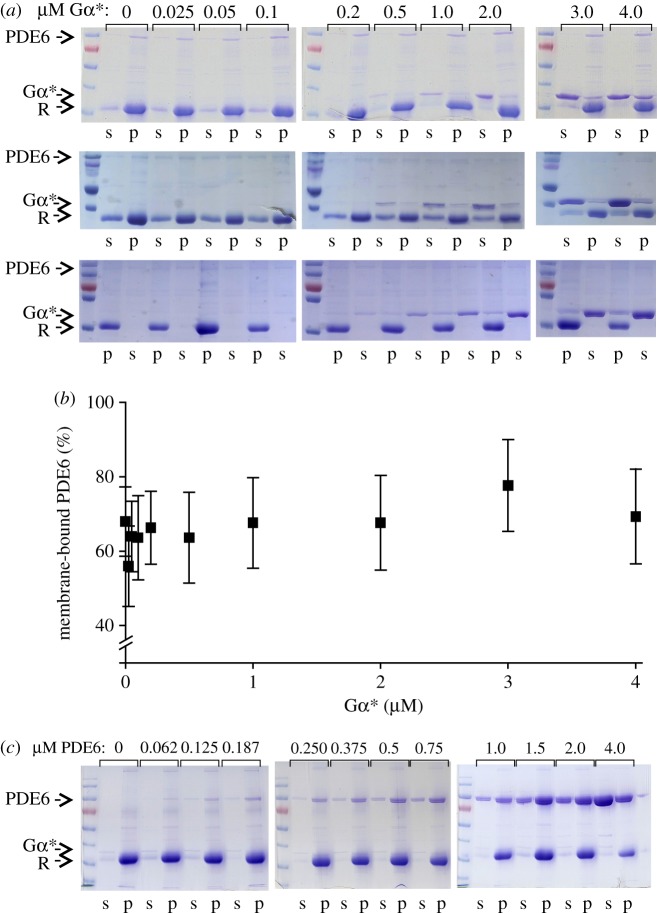
Membrane binding of PDE6. (*a*) Coomassie-stained gels for the three sets of samples used in the PDE6 activity measurements (figure 1*a*; electronic supplementary material, figure S1*b*). Titration of PDE6 (0.1 µM) with Gα* in the presence of disc membranes (10 µM rhodopsin; R). Samples were partitioned into soluble (s) and pellet (p) fractions by centrifugation. (*b*) Densitometric quantification of membrane-bound PDE6 using the gels shown in (*a*). PDE6 (66 ± 3%) was membrane bound on average. Note that, due to the high membrane concentration used in these experiments, the amount of PDE6 bound to the membrane did not depend significantly on the Gα* concentration, which is consistent with a previous study [33]. (*c*) Coomassie-stained gels of a representative set of samples used in the PDE6 activity measurements (figure 1*c*). Titration of Gα* (0.25 µM) with PDE6 in the presence of disc membranes (10 µM rhodopsin; R). Samples were partitioned into soluble (s) and pellet (p) fractions by centrifugation.

The rate of PDE6-catalysed cGMP hydrolysis resulting from titration of PDE6 with Gα* in the presence of membranes (figure 1*a*,*b*) was numerically fitted using Scientist software (MicroMath). Two different activation models were applied (scheme 1). In model 1 (independent activation), membrane-associated PDE6 (PDE6_m_) is assumed to comprise two independent and non-identical Gα*-binding sites (sites 1 and 2; scheme 1*a*) with different affinities for Gα* (*K*_d1_ and *K*_d2_). Occupancy of each site on PDE6_m_ by Gα* leads to formation of Gα* · PDE6_m_ (site 1) and PDE6_m_ · Gα* (site 2), respectively, and induces the cGMP hydrolytic activity of the respective PDE6 catalytic subunit (*k*_cat1_ or *k*_cat2_). Because only 66% of PDE6 is bound to the membranes under the experimental conditions (figure 2*b*), activation of PDE6_s_ is also taken into account.

The concentrations of Gα* · PDE6_m_, PDE6_m_ · Gα* and active PDE6_s_ (PDE6_s_*) as a function of added Gα* were numerically calculated in the fitting procedure (see electronic supplementary material, appendix S1). The rate of PDE6-catalysed hydrolysis of cGMP (*v*_cG_) was then calculated by the following equation:2.2
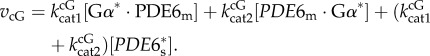


Because the PDE6 activity measured with 8-Br-cGMP reflects the binding of only one Gα* to PDE6 (see §3.2), the rate of 8-Br-cGMP hydrolysis (*v*_BrcG_) was calculated by the following equation:2.3
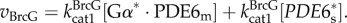


The data points for the Gα* titration experiments performed with cGMP (figure 1*a*) and with 8-Br-cGMP (figure 1*b*) were simultaneously fitted with equations (2.2) and (2.3) using the same set of protein concentrations and dissociation constants but with individual turnover numbers (*k*_cat_). The fit yields the enzymatic parameters summarized in the electronic supplementary material, table S1, though with undefined errors. To estimate the errors in the parameters, a factor *γ* (0 < *γ* < 1) was introduced, which relates 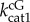 and 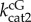 to the overall hydrolytic activity 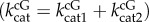 of PDE6:2.4
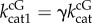
and2.5
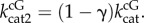


Substitution in equation (2.2) yields2.6
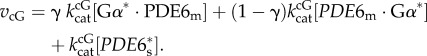


To reduce the number of variables, the data were fitted again with equations (2.6) and (2.3) and with fixed values of γ to yield the enzymatic parameters summarized in the electronic supplementary material, table S2. The resulting titration curves are plotted in the electronic supplementary material, figure S1*b*,*c*.

In model 2 (interdependent activation; scheme 1*b*), the affinities of the two Gα*-binding sites on PDE6_m_ for the first Gα* (*K*_d1_) are assumed to be identical. Formation of Gα* · PDE6_m_ is accompanied by conformational changes that lead to partial cGMP hydrolytic activity 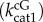 and altered affinity for the second Gα* (*K*_d2_). Binding of the second Gα* to Gα* · PDE6_m_ induces full PDE6 cGMP catalytic activity 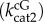. With 8-Br-cGMP as a substrate, we assume that Gα* · PDE6_m_ and Gα* · PDE6_m_ · Gα* have identical hydrolytic activities 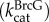.

The concentrations of Gα* · PDE6_m_, Gα* · PDE6_m_ · Gα* and active PDE6_s_ (PDE6_s_*) as a function of added Gα* were numerically calculated in the fitting procedure (see electronic supplementary material, appendix S1) and the rate of PDE6-catalysed hydrolysis of cGMP (*v*_cG_) and 8-Br-cGMP (*v*_BrcG_) was then calculated by the following equations:2.7
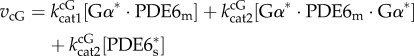
and2.8
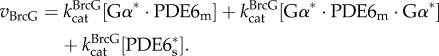


The data points for the Gα* titration experiments performed with cGMP (figure 1*a*) and with 8-Br-cGMP (figure 1*b*) were simultaneously fitted with equations (2.7) and (2.8).

### 2.5. Particle-based reaction–diffusion and ordinary differential equation simulations

**Scheme 2.**
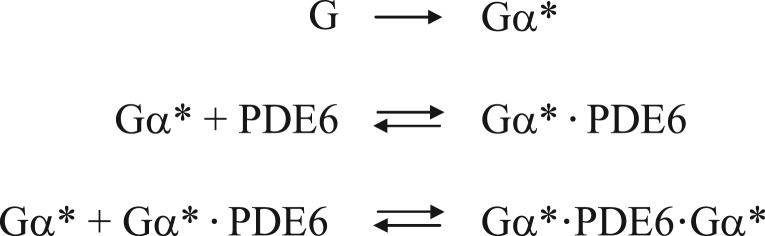
Reactions used in the PBRD and ODE simulations.

Particle-based reaction–diffusion (PBRD) simulations were performed with ReaDDy software [34]. R*, Gα*, PDE6, Gα* · PDE6 and Gα* · PDE6 · Gα* were simulated as explicit space-excluding spherical particles that diffuse on a two-dimensional (2D) disc membrane of area *A* = 1 µm^2^. Initial particle numbers were 250 PDE6, 0 Gα* · PDE6, 0 Gα* · PDE6 · Gα*, 2500 inactive G proteins and 0 Gα*. Inactive G protein particles switched into their active form Gα* with a rate of 1000/s either at random times (noise scenario), or a single R* sequentially created Gα* (signal scenario). No shut-off reactions were included, i.e. R* and Gα* remained active during the 100 ms reaction–diffusion simulation. All particles were uniformly distributed initially and underwent Brownian motion with diffusion constants derived from their size. Physiological particle radii were taken from crystal structures and our cryo-EM data for PDE6 (see electronic supplementary material, appendix S3 for detailed parameter derivation). If particles collided, they were able to undergo reactions based on scheme 2. Reaction rates were parametrized based on the measured kinetic parameters (electronic supplementary material, table S3). Ordinary differential equation (ODE) simulations were conducted with Mathematica 9.0.1.0 and used the same kinetic parameters as in the PBRD signal scenario (see electronic supplementary material, appendix S3 and table S2 for details).

### 2.6. Electron microscopy and image processing

Negative stain EM was performed essentially as described previously [35]. In brief, PDE6 samples were adsorbed onto freshly glow-discharged holey grids (Quantifoil, Germany) covered with an additional thin continuous carbon layer. After negative staining with 2% uranyl acetate, transmission electron microscopic images were collected on a Tecnai G2 Spirit microscope (FEI) operated at 120 kV, which was equipped with a 2 k × 2 k Eagle CCD camera. Micrographs were acquired at a nominal defocus of 1.0–3.5 µm at a nominal magnification of 42 000× using the Leginon system for automated data collection [36]. Particle images were either manually identified or detected semi-automatically. Reference-free class averages were generated using SPIDER [37] or ISAC [38]. Class-averages were then used to generate template three-dimensional (3D) structures in EMAN2 [39]. For cryo-electron microscopy, tPDE6 supplemented with 1% CHAPS was applied to freshly glow-discharged holey grids with an additional thin continuous carbon layer and flash-frozen in liquid ethane using a semi-automated Vitrobot plunger (FEI). Micrographs were collected under the same conditions as for the negative stained samples, but using a cryo-capable holder (Gatan, model 626) and a defocus range of 1.0–5.0 µm. Manually identified particle images were subjected to multiple rounds of multi-reference template matching and 3D *K*-means-like clustering [40] using the negative stain structure as the seeding template. The final cryo-EM density map has been deposited with the EMDB (accession no. EMD-0102). The resulting PDE6 atomic model is based on rigid-body docking of individual domains of the initial homology model obtained by Zeng-Elmore *et al.* [41] into our electron density map (for details see electronic supplementary material, appendix S2).

## 3. Results

### 3.1. Experimental strategy for the enzymatic analysis

The holo-PDE6 (PDE6αβγγ) comprises two catalytic cGMP-binding sites that are activated by Gα* binding to two regulatory sites. This complex architecture of the enzyme allows for a variety of possible activation mechanisms: the two Gα* binding sites could potentially be functionally identical, non-identical or cooperative, and in addition, the two catalytic sites might be identical or non-identical. Furthermore, it is well accepted that the activation mechanism of PDE6 in solution differs from that of the membrane-associated enzyme (see Discussion). We therefore analysed activation of the physiologically relevant membrane-associated PDE6 by using the following experimental strategy:

(i) A classical real-time pH change assay [31] was employed to titrate PDE6 activity with permanently activated Gα-subunits (GαGTPγS, Gα*), i.e. without the normal physiological inactivation of Gα* by its intrinsic GTPase activity (figure 1*a*,*b*).
(ii) The measurements were conducted at high concentrations of PDE6 and membranes to enhance membrane association of PDE6.
(iii) The fraction of soluble and membrane-bound PDE6 in the samples used for enzymatic measurements was quantified by centrifugal pull-down analysis (figure 2). In the numerical analysis of the Gα*-titration curves, the activity of both membrane-associated and soluble PDE6 was taken into account.
(iv) In addition to the native substrate cGMP, we applied 8-bromo-cGMP (8-Br-cGMP), which has been reported to be slowly hydrolysed by toad PDE6 [32]. The striking difference between the two resulting Gα*-titration curves allowed us to discriminate between partially (Gα* · PDE6) and fully activated PDE6 (Gα* · PDE6 · Gα*) in the numerical analysis (see below).
(v) To quantify the enzymatic parameters of membrane-bound PDE6, the two Gα* titration curves obtained with cGMP and 8-Br-cGMP, under otherwise identical experimental conditions, were simultaneously analysed by a numerical fitting procedure using the same set of protein concentrations and dissociation constants but with different activities for cGMP and 8-Br-cGMP.

### 3.2. Binding of the first Gα* to membrane-bound phosphodiesterase 6 induces negligible cGMP hydrolytic activity

In agreement with the literature (see [42,43]), the Gα*-stimulated activation of PDE6 in solution is very inefficient. Under these conditions, titration of PDE6 activity with Gα* resulted in a hyperbolic saturation curve (figure 1*a*; electronic supplementary material, figure S1*a*) with an apparent *K*_d_ of about 20 µM. The shape of the titration curve and the low affinity of Gα* for soluble PDE6 are consistent with an activation mechanism in which interaction between Gα* and soluble PDE6 is only transient, because the two Gα*s, each in complex with a PDE6*γ* subunit, dissociate from the active catalytic PDE6αβ dimer (see Discussion for details). By contrast, in the presence of disc membranes, the Gα* titration assay (figure 1*a*) showed a saturation curve of quite different form, with an initial sigmoidal rise that is clearly apparent in the inset, though difficult to see on the scale of the main panel (see also electronic supplementary material, figure S1*b*). We interpret the sigmoidal shape to indicate that the activation of PDE6 occurs either by cooperative Gα* binding or by successive occupation of two non-identical but independent Gα*-binding sites.

Gα* titration of PDE6 activity in the presence of membranes, using 8-Br-cGMP instead of cGMP as a substrate, resulted in a fundamentally different saturation curve (figure 1*b*). The curve for hydrolysis of 8-Br-cGMP by PDE6 did not exhibit a sigmoidal shape and saturated at about 10 times lower Gα* concentrations. The shallow increase at higher Gα* concentrations (greater than 0.5 µM) can be attributed to the small fraction of soluble PDE6 present in the samples (figure 2) that is activated with low affinity (figure 1*a*). The striking difference in the shape of the Gα* titration curve obtained for 8-Br-cGMP (figure 2*b*) compared with that for cGMP (figure 2*a*) allows us to conclude, firstly, that the hydrolytic activity of the PDE6 is determined not solely by the binding of Gα*, but that the activity is additionally determined in an important manner by the nature of the substrate being hydrolysed. Secondly, the finding that PDE6 activity saturates at 10 times lower Gα* concentration with 8-Br-cGMP as a substrate compared with cGMP strongly suggests that (i) the activity measured with 8-Br-cGMP reflects the binding of only the first Gα* to PDE6, (ii) the activity measured with cGMP reflects binding of both the first and the second Gα*s to PDE6 and (iii) the interaction of PDE6 with the first Gα* occurs with higher affinity than its interaction with the second Gα*.

These concepts can potentially be described by two alternate models of activation (scheme 1): either an ‘independent activation model’ that assumes independent activation of two intrinsically different PDE6α and PDE6β subunits, or an ‘interdependent activation model’ which invokes cooperative activation of two initially equivalent Gα*-binding sites on PDE6. To quantify the affinities of Gα* for the two binding sites on PDE6, as well as the enzymatic activities of PDE6 evoked by binding of one and two Gα*s, we applied a simultaneous fit of the data points obtained with cGMP and 8-Br-cGMP, respectively, using the two models. Importantly, the quantification of PDE6 membrane association by pull-down analysis of all samples used for the activity measurements (figure 2) allowed us to account for the enzymatic activity of soluble and membrane-associated PDE6 within the fitting procedure (see Material and methods, and electronic supplementary material, appendix S1 for details). The two models yielded fits (solid curves) that are indistinguishable on the scale of figure 1*a*,*b*, and that describe the data extremely well. The resulting enzymatic parameters are summarized in table 1. Taken together, the results show that occupancy of the high-affinity Gα*-binding site (model 1) or binding of the first Gα* (model 2) on membrane-associated PDE6 by Gα* (*K*_d1_ < 20 nM) induces full 8-Br-cGMP hydrolysis but very low cGMP hydrolysis (less than 2.5% of maximum). In striking contrast, occupancy of the low-affinity Gα*-binding site (model 1) or binding of the second Gα* (model 2) to membrane-associated PDE6 (*K*_d2_ = 600 nM) induces full cGMP hydrolysis but no further 8-Br-cGMP hydrolysis (table 1).

**Table 1.** Enzymatic parameters (dissociation constants for Gα* (*K*_d_) and maximum rate (turnover number, *k*_cat_)) of membrane-bound PDE6 (see Material and methods, and electronic supplementary material, tables S1 and S2 for details).

	*K*_d_ for Gα* (nM)	*k*_cat_ (cGMP/s)	*k*_cat_ (8-Br-cGMP/s)
Gα* binding to site 1 (model 1) or binding of first Gα* (model 2)	<20	<70	45 ± 10
Gα* binding to site 2 (model 1) or binding of second Gα* (model 2)	600 ± 30	2750 ± 30	0^a^

^a^For model 2, it is assumed that no additional 8-Br-cGMP hydrolytic activity is evoked upon binding of a second Gα*, i.e. that Gα* · PDE6 · Gα* has the same activity as Gα* · PDE6 for 8-Br-cGMP.

The validity of the model was further assessed by measuring cGMP hydrolysis with increasing PDE6 concentration but at a fixed Gα* concentration in the presence of membranes. At low PDE6 concentrations, the resulting hydrolytic activity increased with increasing PDE6, but at higher PDE6 concentration it dramatically decreased (figure 1*c*). The observed shape of the curve, with its decline at high PDE6 concentrations, provides strong qualitative support for our proposed activation mechanism, as follows. At low PDE6 concentrations, Gα* is in excess and occupies both binding sites on PDE6, thereby efficiently activating the enzyme. But when there is substantial excess of PDE6 over Gα*, it is primarily the high-affinity sites that are occupied, which leads to formation of the 1 : 1 Gα* · PDE6 complex at the expense of Gα* · PDE6 · Gα*. In our view, the observed pronounced drop of enzymatic PDE6 activity under these conditions can only be explained by a considerably lower hydrolytic activity of Gα* · PDE6 when compared with Gα* · PDE6 · Gα*. Although it would be interesting to undertake a quantitative evaluation of the effect, this is not currently possible, because the membrane association of PDE6 depends strongly on its concentration (figure 2*c*).

### 3.3. Reaction–diffusion simulation of phosphodiesterase 6 activation

**Figure 3.**
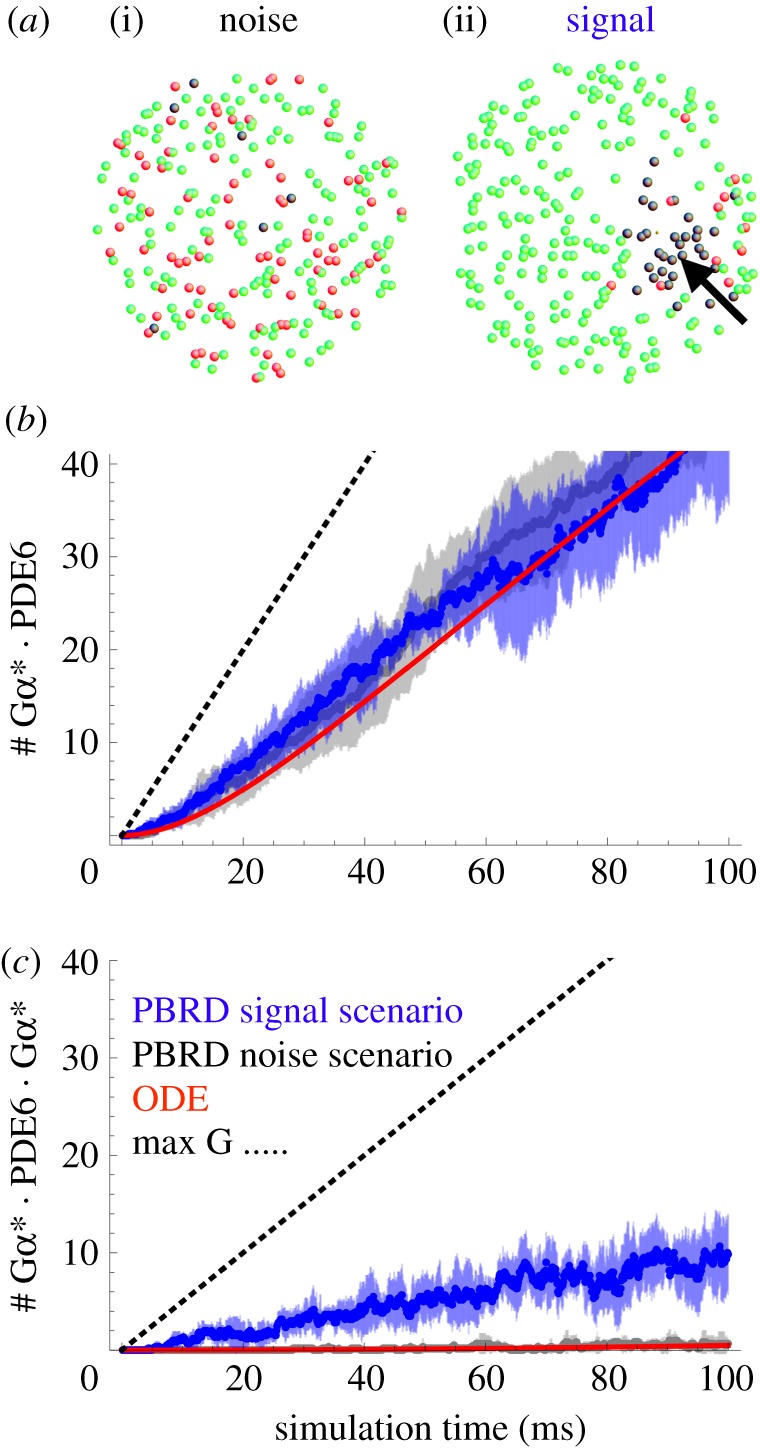
Comparison of the delocalized noise-like and localized signal scenarios by PBRD simulations. (*a*) Two scenarios are compared: a ‘noise’-like scenario (i), in which Gα* arises randomly in time and with uniform spatial distribution across the disc, and a ‘signal’ scenario (ii), in which Gα* is produced locally by one activated rhodopsin molecule (thick arrow indicates initial rhodopsin location). A disc membrane (1 µm^2^) with PDE6 (green), Gα* · PDE6 (red) and Gα* · PDE6 · Gα* (black) is depicted after a 100 ms reaction–diffusion simulation of Gα* production and PDE6 activation (inactive rhodopsins and inactive G proteins are omitted for clarity). (*b*,*c*) PBRD simulations were conducted to compare the kinetics of noise (grey) and signal (blue) scenarios. For each scenario, the time evolution of Gα* · PDE6 (*b*) and Gα* · PDE6 · Gα* (*c*) was computed for six independent simulations (averages are enveloped by standard deviation). The solutions of an ODE model that is spatially indifferent and hence represents the noise scenario are depicted in red. The dotted line indicates the maximum values that could be produced for each quantity, being limited by the supply of Gα*. Note the opposite behaviour of the two scenarios: the noise scenario predominantly produces Gα* · PDE6, while the signal scenario predominantly produces Gα* · PDE6 · Gα* (see electronic supplementary material, movie S1).

To model the spatio-temporal activation pattern on a native disc membrane resulting from sequential PDE6 activation, we conducted PBRD simulations, similar to Schöneberg *et al.* [44], using ReaDDy software [34]. We evaluated PDE6 activation by Gα* in response to two system inputs: (i) a ‘noise’-like activation scenario, in which Gα* is spontaneously produced and thus is uniformly distributed on the disc membrane, and (ii) a ‘signal’ scenario, in which Gα* is produced locally by a single active R* molecule (figure 3*a*). Both scenarios were simulated with the same initial disc membrane topology, consisting of a 2D disc membrane (*A* = 1 µm^2^) that was uniformly populated with 250 PDE6 particles. R*, PDE6 and Gα* were simulated as explicit particles that undergo 2D diffusional motion on the disc membrane. For the signal scenario, we used a Gα* production rate of 1000/s/R*, which is roughly the speed of the reaction under assumed physiological conditions of the rod cell [29]. The Gα*/PDE6 binding affinities determined above (table 1) result in the constraints *K*_d1_ = *k_−_*_1_/*k*_1_ and *K*_d2_ = *k*_−2_/*k*_2_ (see electronic supplementary material, appendix S3). Other parameters such as diffusion coefficients were determined as described in the Material and methods, and electronic supplementary material, appendix S3. Six PBRD runs of 100 ms were performed independently in order to compute means and standard deviations of their activation kinetics. We also conducted ODE simulations (see Material and methods and electronic supplementary material, appendix S3 for details) with the same kinetic parameters, for comparison with the PBRD signal scenario.

When Gα* production occurs in a delocalized way, by spontaneous activation (low Gα* density, PBRD noise scenario), the form of PDE6 that is created is predominantly the singly bound Gα* · PDE6 (rather than the doubly bound Gα* · PDE6 · Gα*) across the entire disc during the time course of the simulation (100 ms; figure 3*b* and electronic supplementary material, figure S4). High-affinity binding of Gα* to PDE6 acts here to sequester Gα* and thereby prevent the activation of PDE6 by low-affinity binding of a second Gα* to Gα* · PDE6. Note that the rate of spontaneous Gα* production that we have adopted in the noise scenario is very high, and presumably far exceeds the rate of spontaneous Gα* formation *in vivo*. We chose this high rate in order to obtain the same overall rate of Gα* production as in the signal scenario and thus to have a rigorous test of the influence of uniform (noise scenario) versus local (signal scenario) Gα* production on PDE6 activation. Even with a spontaneous Gα* activation rate as high as 1000/s (i.e. by a factor 10^4^ faster than in the physiological context; see Discussion), the level of fully active Gα* · PDE6 · Gα* does not rise above 0.5 molecules µm^−2^ on average during the first 100 ms. It is notable that the results of the ODE simulations, in which Gα* is activated by R*, are very similar to PBRD simulations of the noise scenario (figure 3*b*; electronic supplementary material, figure S4). This is because the ODE method is inherently spatially indifferent, i.e. it simulates a well-mixed and equilibrated system (see [44] for a thorough discussion). However, when the same amount of Gα* is not uniformly distributed but instead produced locally by a single active rhodopsin (high local Gα* density and PBRD signal scenario), the active Gα* · PDE6 · Gα* form dominates 10-fold over Gα* · PDE6 throughout the first 100 ms.

### 3.4. The structure of the active phosphodiesterase 6αβ dimer features mobile catalytic domains

**Figure 4.**
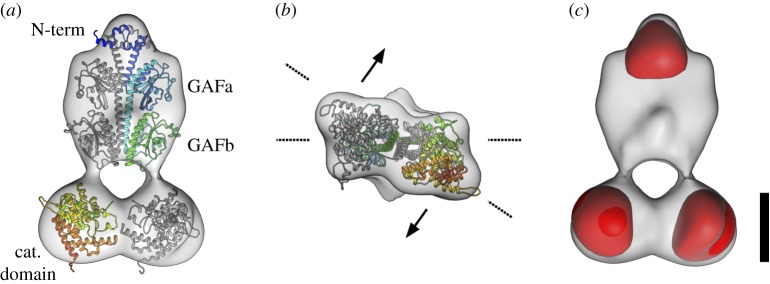
Cryo-EM structural characterization of PDE6. (*a*) Front view of the electron density map of the final tPDE6 cryo-EM structure (grey), with a rigid-body docked homology model of bovine PDE6. The α-chain is depicted in a rainbow gradient from N-terminus to C-terminus, while the β chain is presented in grey. (*b*) View from the catalytic domain of tPDE6. The catalytic domain is tilted out of plane with respect to the plane spanned by the GAFa/b domains. The overall model thus assumes a twisted topology and the functional regions of the catalytic domain face in different directions (exemplified by arrows). (*c*) Electron density map of tPDE6 together with the remaining 3D variability (red) as estimated from the aligned particle images. The dominant variability localizes to the catalytic domains and the N-terminal feature. Scale bar is 50 Å.

Negative-stain EM structures of PDE6 [20–22] and the recent cryo-EM structure [23] agree on a general side-by-side, elongated arrangement of the PDE6 *α* and β chains. Similarly, our initial common line-based negative-stain structure of bovine holo-PDE6 (PDE6αβγγ, electronic supplementary material, figure S3*a–c*) resembles the flat, bell-shaped structures published earlier [20,21,23]. As in the published structures, we observe two regions of low electron density that have the appearance of cavities. Based on the mass distribution in the structure, the larger cavity appears to be separating the catalytic domains from the GAF domains, while the smaller cavity appears to be situated between the four GAF domains. Since the negative-stain procedure is known to be prone to structural artefacts [35], we strived to validate the structure of PDE6 by cryo-EM. To overcome the preferential orientation of holo-PDE6 on the carrier grid surface, we generated a truncated PDE6 by limited trypsination. Proteolysis removes approximately 1 kDa of the C-termini of both PDE6 catalytic subunits with their lipid moieties and also the two inhibitory PDE6γ subunits [9]. The resulting catalytic core (tPDE6) has been demonstrated to be an active and soluble enzyme [9]. Our results differed from those of Zhang *et al.* [23], but agree with Kajimura *et al.* [21] in showing the PDE6*γ*-free tPDE6 to retain the bell-shaped overall structure of the holoenzyme (see electronic supplementary material, appendix S2 for discussion). Consistent with a recent cross-linking study [41], the cryo-EM structure shows the functional sites of the PDE6 situated on opposing faces of the enzyme (figure 4*b*).

Intriguingly, during our reconstruction, we observed strong 3D variability [45] for the catalytic domains (figure 4*c*). This implies a flexibility of the catalytic domain with respect to the N-terminal half of tPDE6, which is different to the case of the Fab-bound catalytic domains that were found by Zhang *et al.* [23] to be in a fixed orientation relative to GAFa. Based on atomic models of a nearly full-length PDE2 crystal structure [19] and a recent homology model of PDE6 [41], we derived a new PDE6 model, by sequential rigid-body fitting (see electronic supplementary material, appendix S2 for details), that agrees well with our experimental electron density map (figure 4*a*,*b*).

## 4. Discussion

### 4.1. Asymmetric activation of membrane-associated phosphodiesterase 6

The classical way to study PDE6 activation *in vitro* is the titration of enzymatic activity with Gα*. When performed in solution, the resulting dose–response curves consistently reveal that Gα* has a very low affinity for soluble PDE6 (figure 1*a*; electronic supplementary material, figure S1*a*). This finding is readily explained by the fact that activation of PDE6 in solution involves successive Gα*-mediated dissociation of the two inhibitory PDE6*γ* subunits from the catalytic PDE6αβ dimer and thus formation of partially active PDE6αβγ and fully active PDE6αβ (reviewed in [42,43]). Together with results obtained by a reverse titration, i.e. inhibition of a fully active, truncated PDE6αβ (tPDE6; obtained by limited proteolysis of PDE6αβγγ) with exogenous PDE6γ, it was concluded that partially activated PDE6αβγ has 50% of the hydrolytic cGMP activity of the fully activated PDE6αβ in solution (see [17]).

In the presence of membranes, however, the affinity of Gα* for PDE6 is dramatically enhanced, as it forms membrane-associated Gα* · PDE6 and Gα* · PDE6 · Gα* complexes (reviewed in [42,43]). Under these conditions, and with mammalian PDE6, the Gα* titration curves are biphasic or sigmoidal, which has led to conflicting interpretations. Proposed activation mechanisms include inhomogeneous populations of Gα* or PDE6, non-identical Gα*-binding sites [27,46–48] and cooperative binding [49,50]. There is also substantial disagreement regarding the relative hydrolytic activity of the intermediary 1 : 1 Gα* · PDE6 complex; estimates range from less than 20% [27,46,51,52] up to greater than 80% [49] of maximum PDE6 activity.

Our finding that the PDE6 activity measured with the cGMP analogue 8-Br-cGMP monitors formation of Gα* · PDE6 allows us to dissect the activation mechanism. By employing 8-Br-cGMP in parallel to the native substrate, cGMP, we can now quantify the affinity of Gα* for the two binding sites on the membrane-bound PDE6, as well as the enzymatic activity of the membrane-associated, intermediary 1 : 1 Gα* · PDE6 complex. The results show that the activation of PDE6 by Gα* in the presence of membranes is a sequential two-step process: the first Gα* interacts with PDE6 with high affinity but induces only negligible cGMP hydrolytic activity, and this is followed by the binding of a second Gα* to a low-affinity site, which leads to full cGMP hydrolytic activity. It is noteworthy that the titration curve of a kinetic light-scattering signal, which was assigned to be a monitor of the interaction of the first Gα* with membrane-bound PDE6 and that was shown to saturate at approximately 10% of the maximal hydrolytic activity [27], can now be seen to reflect the high-affinity binding measured in our biochemical assay using 8-Br-cGMP (figure 1*b*).

In the following, we will first discuss the implications of the asymmetric PDE6 activation mechanism on visual signal transduction and later the mechanistic basis of asymmetric PDE6 activation.

### 4.2. Role of phosphodiesterase 6 functional asymmetry in phototransduction

The data of this study shine new light on the intriguing capacity of rod photoreceptor cells to detect single quanta of light. A high amount and high density of the signalling proteins (rhodopsin, G protein and PDE6) is required to detect and rapidly transmit the light signal. But all these proteins are prone to produce background noise originating from spontaneous activation events, and the dominant source of such noise will determine the threshold for detection of a photon [53]. Thermal activation of rhodopsin is observed in rare spontaneous bumps of membrane current [54,55]. In addition, electrophysiological recordings show a continuous component of noise that is attributed to spontaneous PDE6 activation, but no detectable component of the continuous noise could be attributed to the spontaneous activation of G protein [56]. The intrinsic nucleotide exchange rate of rod G protein is about 10^−4^ s^−1^ *in vitro* at 37°C [57]. Given the slow GTPase activity of free Gα* (reviewed in [42]), each spontaneously activated copy of Gα* would eventually bind to PDE6. If each such Gα* activated one subunit of the PDE6, then this would generate a large and easily detectable noise component (see [58]). Why is such noise not observed? We believe that the reason lies in the activation mechanism of the membrane-bound, mammalian rod PDE6, whose hydrolytic activity is only appreciably triggered when two copies of Gα* are simultaneously bound to the same PDE6 molecule. The reaction–diffusion simulations have shown that at the low Gα* density that prevails in the absence of activated rhodopsin (noise scenario in figure 4), the high-affinity binding of Gα* to PDE6 acts to sequester Gα* in a functionally inactive form. Thus, Gα* · PDE6 with no significant hydrolytic activity is exclusively formed under physiological conditions in the dark. However, at the high local concentration of Gα* generated by an active rhodopsin molecule (signal scenario in figure 4), the simultaneous occupancy of two sites on the PDE6 by Gα* is a frequent event, leading to the rapid activation of the enzyme and thereby to sufficient cGMP hydrolysis to trigger neuronal signalling. Such a coincidence mechanism [59] of PDE6 activation makes the G protein/effector pair noise-resistant, but allows a fast response to the light signal. A significant PDE6 activity is only achieved with a high local density of Gα*, as is produced by the local activation originating from active rhodopsin. Consistent with such a coincidence or density switch, electrophysiological recordings of mammalian rod photoreceptor cells have recently suggested a significantly lower hydrolytic activity for spontaneously activated PDE6 when compared to the light activated enzyme [24]. Interestingly, in retinal cone cells, the catalytic subunits of PDE6 are identical and thus likely symmetric in their activation, which would be consistent with the higher noise observed in cones compared with rods [60]. We note that activation density switches are also found in other biological systems such as the highly cooperative Ca^2+^-sensor synaptotagmin in neurotransmission. In that case, the crucial factor is a high activation density of Ca^2+^ flowing through Ca^2+^-channels localized around active zones in presynaptic neurons [61].

**Figure 5.**
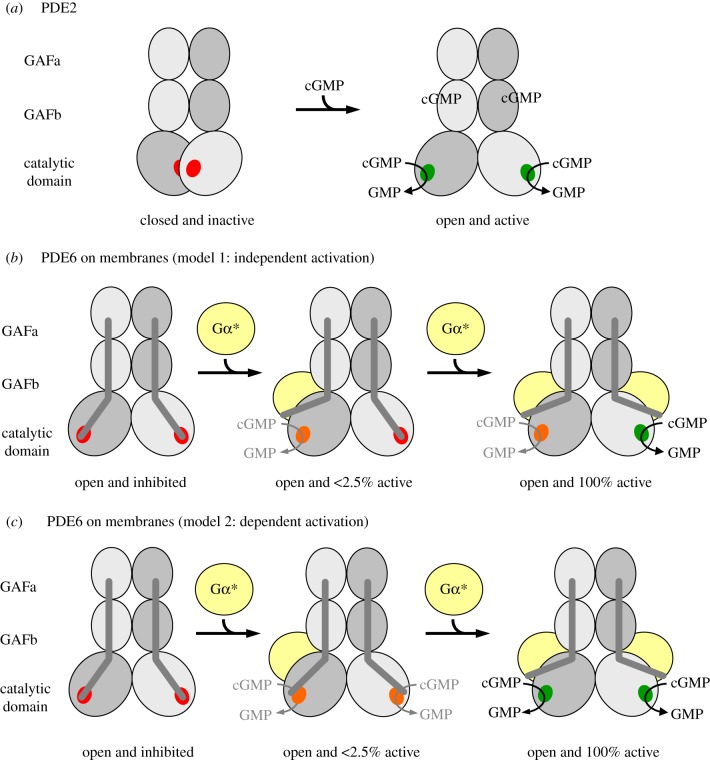
Models of PDE activation. Each catalytic PDE subunit consists of two GAF domains and one catalytic domain. The three domains of each catalytic subunit are arranged in a crossover architecture (see figure 4*a* and electronic supplementary material, appendix for details). (*a*) Activation of PDE2 as proposed by Pandit *et al.* [19]: in the closed, inactive PDE2 conformation, access of the substrate to the catalytic cGMP-binding sites (red) is blocked by mutual inhibition of the catalytic domains. Cooperative binding of cGMP to the GAFb domains induces outward rotation of the catalytic domains and hence formation of the open, active PDE2 conformation. (*b*,*c*) Activation of membrane-associated rod PDE6: the PDE6αβ-dimer adopts an open conformation, which is inhibited by tight interaction with two PDE6γ subunits (dark-grey rods) in the resting state. Activation of membrane-associated PDE6 is due to removal of inhibition following the successive binding of two Gα*s (membrane is omitted for clarity). The catalytic cGMP-binding sites are either fully inhibited (red), or have a very low (less than 2.5% of maximum; orange) or high hydrolytic cGMP activity (green). In model 1 (*b*), the two catalytic PDE6 subunits are intrinsically different with respect to their affinities for Gα* and their catalytic activity, respectively, and are independently activated by Gα*. Occupancy of the high-affinity Gα*-binding site induces very low cGMP hydrolytic activity, whereas occupancy of the low-affinity Gα*-binding site induces full cGMP hydrolytic activity. In model 2 (*c*), the two catalytic PDE6 subunits are functionally equal. High-affinity binding of the first Gα* to either of the two binding sites on the PDE6 induces a conformational change that confers very low activity to both catalytic sites. Full activation of both catalytic subunits requires low-affinity binding of a second copy of Gα*.

The PDE6 activation mechanism described here may also have consequences for the termination of rod phototransduction. It is well accepted that the falling phase of the rod photocurrent is limited by the lifetime of active PDE6 [62]. If the interdependent model of PDE6 activation applies (figure 5*c*; see Discussion below) deactivation of only one Gα* by its GTPase activity and subsequent release of Gα from a given fully activated PDE6 enzyme would suffice to deactivate its enzymatic activity. This mechanism would thus translate into an accelerated termination the photoresponse in rod cells. For a more thorough analysis of the implications of an asymmetric PDE6 activation for rod phototransduction, see Lamb *et al.* [58].

### 4.3. Mechanistic basis of asymmetric phosphodiesterase 6 activation

Pandit *et al.* [19] suggested an activation mechanism of PDE2 in which both catalytic subunits rotate outwards upon cGMP binding to the GAFb domains (figure 5*a*). We suggest that our cryo-EM structure of pre-activated tPDE6αβ is analogous to this putative active, ‘open’ conformation of PDE2 [19] (figure 5*a*). Notably, our active tPDE6αβ structure is essentially identical to the inactive PDE6αβγγ structure obtained by Zhang *et al.* [23]. We thus conclude that in PDE6, the catalytic core is always in an open, intrinsically active conformation that is kept inactive by tight binding of the two PDE6-specific inhibitory PDE6γ subunits (see electronic supplementary material, appendix for a detailed discussion).

It is known that activation of membrane-bound PDE6 by Gα* involves displacement of the two PDE6γ subunit C-termini from the catalytic cGMP sites on PDE6αβ [17,18]. However, a conclusive model of the PDE6 activation mechanism is still lacking. Two different classes of model can be envisaged to explain the functional asymmetry of PDE6 described above. Model 1 (independent activation; figure 5*b*) assumes that the two catalytic PDE6 subunits are intrinsically different with respect to substrate specificity and affinity for Gα*, respectively, and that both subunits are independently activated by Gα* binding. According to this model, one catalytic PDE6 subunit binds Gα* with high affinity and is able to hydrolyse only 8-Br-cGMP, and at a low rate, while the other catalytic PDE6 subunit binds Gα* with low affinity and is able to hydrolyse only cGMP, but at a high rate. Although in this model rod PDE6 would retain only half of its potential hydrolytic power, it is, in principle, possible due to the heterodimeric composition of PDE6αβ. Indeed, it has previously been suggested that the two PDE6γ binding sites on PDE6αβ in solution are not identical [63,64] and that only one of the two sites mediates PDE6 inhibition [63]. On the other hand, such asymmetry has not been detected in the majority of studies on proteolytically activated PDE6αβ (tPDE6) (for example, [17]). Furthermore, a study on chimeric homodimeric PDE6 enzymes indicates that the catalytic domains of PDE6α and PDE6β are enzymatically equivalent in solution [65]. An intrinsic functional difference between the two catalytic subunits is thus unlikely, and if it occurred would need to be confined to the membrane-bound and Gα*-activated enzyme.

Although both models fit the data equally well (figure 1*a*,*b*), we thus favour model 2 (interdependent activation, figure 5*c*). According to this model, the two Gα* binding sites of PDE6 are initially equivalent. High-affinity binding of the first Gα* to either one of the two binding sites induces a conformational change that leads to a ‘primed’ Gα* · PDE6 state. This first transition is not enough to uncover the catalytic sites for access by cGMP, but is likely to be accompanied by significant structural rearrangements as indicated by light-scattering changes evoked by interaction of the first Gα* with membrane-bound PDE6 [27] (see below). Full activation of both catalytic subunits requires the low-affinity binding of a second copy of Gα*. To explain the results obtained with 8-Br-cGMP, we hypothesize that this substrate has access to both catalytic sites even in the ‘primed’ Gα* · PDE6 state. Likewise, 8-Br-cGMP has access in the fully active state, and in both configurations the rate of 8-Br-cGMP hydrolysis is equally low. Accordingly, in this model, the two catalytic sites themselves are functionally identical (although differences are not ruled out), yet this mechanism still provides a huge difference in activity towards cGMP between the states with one and two Gα*s bound.

Given the symmetric activation of soluble PDE6, the question arises as to how membrane binding imposes cooperativity in the activation of PDE6 by Gα*. In this regard, a key result of our structural analysis is the strong 3D variability of the catalytic domains (figure 4*c*), implying a flexibility of these C-terminal domains in the active tPDE6. Furthermore, both C-termini of the catalytic subunits with their prenyl membrane anchors are highly flexible, as has directly been visualized in a recent electron microscopy study of native PDE6 by the highly variable location of a prenyl-binding protein (PrBP; that had originally been termed the PDE6*δ* subunit) [28]. It is thus likely that PDE6 is able to adopt multiple orientations on the disc membrane surface. In a tentative model that is consistent with the proposed activation mechanism, we assume that PDE6 preferentially lies flat on the membrane in the inactive resting state. Because the cryo-EM structure shows the functional sites of PDE6 located on opposing faces of the enzyme (figure 4*b*), it is tempting to speculate that this resting orientation of PDE6 only allows initial coupling of Gα* to one binding site. Binding of the first Gα* triggers PDE6 to adopt a more (or even fully) upright orientation without full exposure of either catalytic site. Importantly, such a reorientation of PDE6 would correspond to a mass movement orthogonal to the membrane and would thus nicely explain the increase in light scattering of disc membranes upon binding of the first Gα* to membrane-bound PDE6 [27]. The reorientation on the membrane is proposed to allow binding of the second Gα* with lower affinity under conditions with high local Gα* density, which results in conformational changes that fully expose both catalytic sites, thereby unleashing the full catalytic activity of the enzyme. We emphasize that other structural interpretations such as a Gα*-induced bending of membrane-bound PDE6 are possible. However, the key finding of this study, namely that hydrolytic PDE6 activity is only appreciably triggered when two Gα* molecules are bound to PDE6, is model independent. Irrespective of how the asymmetric activation mechanism is achieved by the membrane-bound PDE6, it provides the rod cell with a coincidence switch that allows for noise filtering at the effector stage in phototransduction. Further implications of this new understanding of PDE6 activation on the predicted electrical responses of rod photoreceptors are provided by Lamb *et al.* [58].

## Supplementary Material

Supplementary Information

## Data accessibility

The final cryo-EM density map has been deposited with the EMDB (accession no. EMD-0102).
