# Supplementary material for: It takes two transducins to activate the cGMP-phosphodiesterase 6 in retinal rods
Source: Open Biol. 2018 Aug 1;8(8):180075. doi: 10.1098/rsob.180075 (PMC6119865; doi:10.1098/rsob.180075)
Supplement: Supplementary Information [file rsob180075supp1.pdf]

# Supplementary Information

## It takes two transducins to activate the cGMP-phosphodiesterase 6 in retinal rods

Bilal M. Qureshi[1¶,#a], Elmar Behrmann[1¶,#b], Johannes Schöneberg[2,#c], Justus Loerke[1], Jörg Bürger[1], Thorsten Mielke[1,3], Jan Giesebrecht[1,#d], Frank Noé[2], Trevor D. Lamb[4], Klaus Peter Hofmann[1,5], Christian M. T. Spahn[1], and Martin Heck[1*]

### S1 Appendix. Numerical fitting procedure of PDE6 activation

The rate of PDE6 catalyzed cGMP hydrolysis resulting from titration of PDE6 with Gα* in the presence of membranes (figures 1*a,b*) were numerically fitted using Scientist software (MicroMath). Two different models were applied, namely an 'independent activation model' that assumes independent activation of two intrinsically different PDE6α and PDE6β subunits (model 1) and an 'interdependent activation model' which invokes cooperative activation of two equivalent Gα* binding sites on PDE6 (model 2). Because only 66 % of PDE6 is bound to the membranes under the experimental conditions (see figure 2*b*), activation of soluble PDE6 ($PDE6_s$) is also taken into account.

*Model 1(independent activation)*

Membrane associated PDE6 ($PDE6_m$) is assumed to comprise two independent Gα*-binding sites (site 1 and site 2) with different affinities for Gα* ($K_{d1}$ and $K_{d2}$). The following dissociation constants are given for the complexes formed by Gα* with PDE6:

Binding of Gα* to site 1 of $PDE6_m$:

$$K_{d1} = \frac{[G\alpha^*][PDE6_{m1}]}{[G\alpha^* \bullet PDE6_m]} \qquad (1)$$

Binding of Gα* to site 2 of PDE6$_m$: $$K_{d2} = \frac{[G\alpha^*][PDE6_{m2}]}{[PDE6_m \bullet G\alpha^*]} \quad (2)$$

Binding of Gα* to either site of PDE6$_s$: $$K_{d3} = \frac{[G\alpha^*][PDE6_s]}{[G\alpha^* \bullet PDE6_s]} \quad (3)$$

The mass balance for Gα∗, PDE6$_{m1}$, PDE6$_{m2}$ and PDE6$_s$ are:

$$[G\alpha^*]_{tot} = [G\alpha^*] + [G\alpha^* \bullet PDE6_m] + [PDE6_m \bullet G\alpha^*] + [G\alpha^* \bullet PDE6_s] \quad (4)$$

$$[PDE6_{m1}]_{tot} = [PDE6_{m1}] + [G\alpha^* \bullet PDE6_m] \quad (5)$$

$$[PDE6_{m2}]_{tot} = [PDE6_{m2}] + [PDE6_m \bullet G\alpha^*] \quad (6)$$

$$[PDE6_s]_{tot} = [PDE6_s] + [G\alpha^* \bullet PDE6_s] \quad (7)$$

Note that [PDE6$_{m1}$]$_{tot}$, [PDE6$_{m2}$]$_{tot}$ and [PDE6$_s$]$_{tot}$ refer to the concentration of individual catalytic subunits of PDE6. Accordingly, [PDE6$_s$]$_{tot}$ is twice the concentration of initially present soluble holo-PDE6.

For numerical computing three additional variables are defined:

$$x_1 = \frac{PDE6_{m1}}{K_{d1}} = \frac{G\alpha^* \bullet PDE6_m}{G\alpha^*} \quad (8)$$

$$x_2 = \frac{PDE6_{m2}}{K_{d2}} = \frac{PDE6_m \bullet G\alpha^*}{G\alpha^*} \quad (9)$$

$$x_s = \frac{PDE6_s}{K_{d3}} = \frac{G\alpha^* \bullet PDE6_s}{G\alpha^*} \quad (10)$$

Using Eqs. 9-11, the variables [Gα*•PDE6$_m$], [PDE6$_m$•Gα*] and [Gα*•PDE6$_s$] can now be expressed as functions of [Gα*]:

$$[G\alpha^* \bullet PDE6_m] = x_1 \cdot [G\alpha^*] \quad (11)$$

$$[PDE6_m \bullet G\alpha^*] = x_2 \cdot [G\alpha^*] \quad (12)$$

$$[G\alpha^*\bullet PDE6_s] = x_s \cdot [G\alpha^*] \quad (13)$$

Substituting for [Gα*•PDE6$_m$], [PDE6$_m$•Gα*] and [Gα*•PDE6$_s$] in the balance equations (Eqs. 4-7) yields:

$$[G\alpha^*] = \frac{[G\alpha^*]_{tot}}{1 + x_1 + x_2 + x_s} \quad (14)$$

$$[PDE6_{m1}] = [PDE6_{m1}]_{tot} - [G\alpha^*\bullet PDE6_m] \quad (15)$$

$$[PDE6_{m2}] = [PDE6_{m2}]_{tot} - [PDE6_m \bullet G\alpha^*] \quad (16)$$

$$[PDE6_s] = [PDE6_s]_{tot} - [G\alpha^*\bullet PDE6_s] \quad (17)$$

Eqs. 8-17 were used for numerical calculation of [Gα*•PDE6$_m$], [PDE6$_m$•Gα*] and [Gα*•PDE6$_s$] as a function of [Gα*]$_{tot}$ in the fitting procedure.

*Model 2 (interdependent activation)*

Membrane associated PDE6 (PDE6$_m$) is assumed to comprise two identical Gα*-binding sites. Binding of the first Gα* to PDE6$_m$ with the affinity $K_{d1}$ results in conformational changes that alters the affinity of the second Gα* ($K_{d2}$). The following dissociation constants are given for the complexes formed by Gα* with PDE6:

Binding of first Gα* to PDE6$_m$: $$K_{d1} = \frac{[G\alpha^*][PDE6_m]}{[G\alpha^*\bullet PDE6_m]} \quad (18)$$

Binding of second Gα* to Gα*•PDE6$_m$: $$K_{d2} = \frac{[G\alpha^*][G\alpha^*\bullet PDE6_m]}{[G\alpha^*\bullet PDE6_m \bullet G\alpha^*]} \quad (19)$$

Binding of Gα* to either site of PDE6$_s$: $$K_{d3} = \frac{[G\alpha^*][PDE6_s]}{[G\alpha^*\bullet PDE6_s]} \quad (20)$$

The mass balance for Gα*, PDE6$_m$, and PDE6$_s$ are:

$$[G\alpha^*]_{tot} = [G\alpha^*] + [G\alpha^* \bullet PDE6_m] + 2[G\alpha^* \bullet PDE6_m \bullet G\alpha^*] + [G\alpha^* \bullet PDE6_s] \quad (21)$$

$$[PDE6_m]_{tot} = [PDE6_m] + [G\alpha^* \bullet PDE6_m] + [G\alpha^* \bullet PDE6_m \bullet G\alpha^*] \quad (22)$$

$$[PDE6_s]_{tot} = [PDE6_s] + [G\alpha^* \bullet PDE6_s] \quad (23)$$

Note that $[PDE6_s]_{tot}$ refers to the concentration of individual catalytic subunits of $PDE6_s$.

For numerical computing three additional variables are defined:

$$x_1 = \frac{G\alpha^*}{K_{d1}} = \frac{G\alpha^* \bullet PDE6_m}{PDE6_m} \quad (24)$$

$$x_2 = \frac{G\alpha^*}{K_{d2}} = \frac{G\alpha^* \bullet PDE6_m \bullet G\alpha^*}{G\alpha^* \bullet PDE6_m} \quad (25)$$

$$x_s = \frac{G\alpha^*}{K_{d3}} = \frac{G\alpha^* \bullet PDE6_s}{PDE6_s} \quad (26)$$

Using Eqs. 24-26, the variables $[G\alpha^* \bullet PDE6_m]$, $[G\alpha^* \bullet PDE6_m \bullet G\alpha^*]$ and $[G\alpha^* \bullet PDE6_s]$ can now be expressed as functions of [PDE6]:

$$[G\alpha^* \bullet PDE6_m] = x_1 \cdot [PDE6_m] \quad (27)$$

$$[G\alpha^* \bullet PDE6_m \bullet G\alpha^*] = x_1 x_2 \cdot [PDE6_m] \quad (28)$$

$$[G\alpha^* \bullet PDE6_s] = x_s \cdot [PDE6_s] \quad (29)$$

Substituting for $[G\alpha^* \bullet PDE6_m]$, $[G\alpha^* \bullet PDE6_m \bullet G\alpha^*]$ and $[G\alpha^* \bullet PDE6_s]$ in the balance equations (Eqs. 21-23) yields:

$$[PDE6_m] = \frac{[PDE6_m]_{tot}}{1 + x_1 + x_1 x_2} \quad (30)$$

$$[PDE6_s] = \frac{[PDE6_s]_{tot}}{1 + x_s} \quad (31)$$

$$[G\alpha^*] = [G\alpha^*]_{tot} - [G\alpha^* \bullet PDE6_m] - 2[G\alpha^* \bullet PDE6_m \bullet G\alpha^*] - [G\alpha^* \bullet PDE6_s] \quad (32)$$

Eqs. 24-32 were used for numerical calculation of [Gα*•PDE6$_m$], [Gα*•PDE6$_m$•Gα*] and [Gα*•PDE6$_s$*] as a function of [Gα*]$_{tot}$ in the fitting procedure.

Figure S1

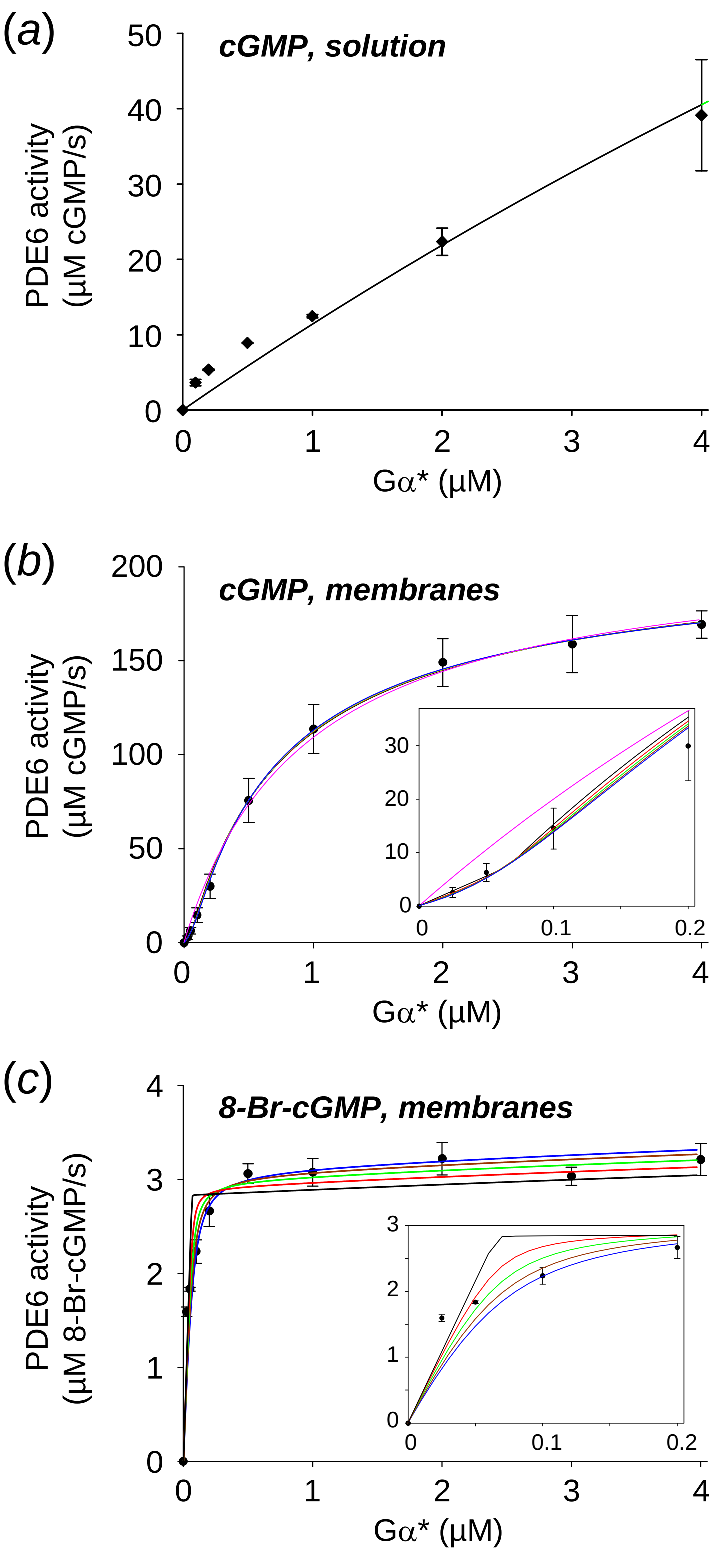

**Figure S1. Quantitative analysis of the enzymatic activity of PDE6.** (*a*) Activity of soluble PDE6 measured as rate of cGMP (same data as in figure 1*a*). Solid line represents best fit to the data points using a hyperbolic function (see Materials and methods for details). (*b,c*) Activity of PDE6 in the presence of disc membranes measured as rate of cGMP (*b*; same data as in figure 1*a*) or 8-Br-cGMP (*c*; same data as in figure 1*b*) hydrolysis at increasing Gα* concentrations. Insets depict the lower concentration range on expanded scales. Solid lines represent best fits to the data points using fixed values for γ (see Materials and methods for details and Table S2): γ = 0 (blue), γ = 0.01 (brown), γ = 0.02 (green), γ = 0.03 (red) and γ = 0.04 (black). The pink line in (*b*) represents a hyperbolic fit to the data.

**Table S1** Dissociation constants for Gα* ($K_d$) and maximum rates (turnover numbers, $k_{cat}$) of membrane bound PDE6 obtained by fitting the experimental data using eqs. (2.2) and (2.3).

| $K_{d1}$ (nM) | $K_{d2}$ (nM) | $k_{cat1}^{cG}$ (cGMP/s) | $k_{cat2}^{cG}$ (cGMP/s) | $k_{cat1}^{BrcG}$ (8-Br-cGMP/s) |
|---|---|---|---|---|
| 19 | 586 | $10^{-14}$ | 2744 | 47 |

**Table S2** Dissociation constants for Gα* ($K_d$) and maximum rates (turnover numbers, $k_{cat}$) of membrane bound PDE6 obtained by fitting the experimental data using eqs. (2.3) and (2.6) and with fixed values of γ.

| γ | $K_{d1}$ (nM) | $K_{d2}$ (nM) | $k_{cat}^{cG} = k_{cat1}^{cG} + k_{cat2}^{cG}$ (cGMP/s) | $k_{cat1}^{Br-cG}$ (8-Br-cGMP/s) |
|---|---|---|---|---|
| 0.04 | 0.1 ± 5 | 664 ± 35 | 2778 ± 38 | 43 ± 11 |
| 0.03 | 3 ± 6 | 640 ± 31 | 2768 ± 34 | 44 ± 10 |
| 0.02 | 8 ± 7 | 620 ± 27 | 2759 ± 31 | 45 ± 9 |
| 0.01 | 14 ± 7 | 602 ± 25 | 2750 ± 29 | 46 ± 9 |
| 0 | 19 ± 8 | 586 ± 23 | 2744 ± 27 | 47 ± 9 |

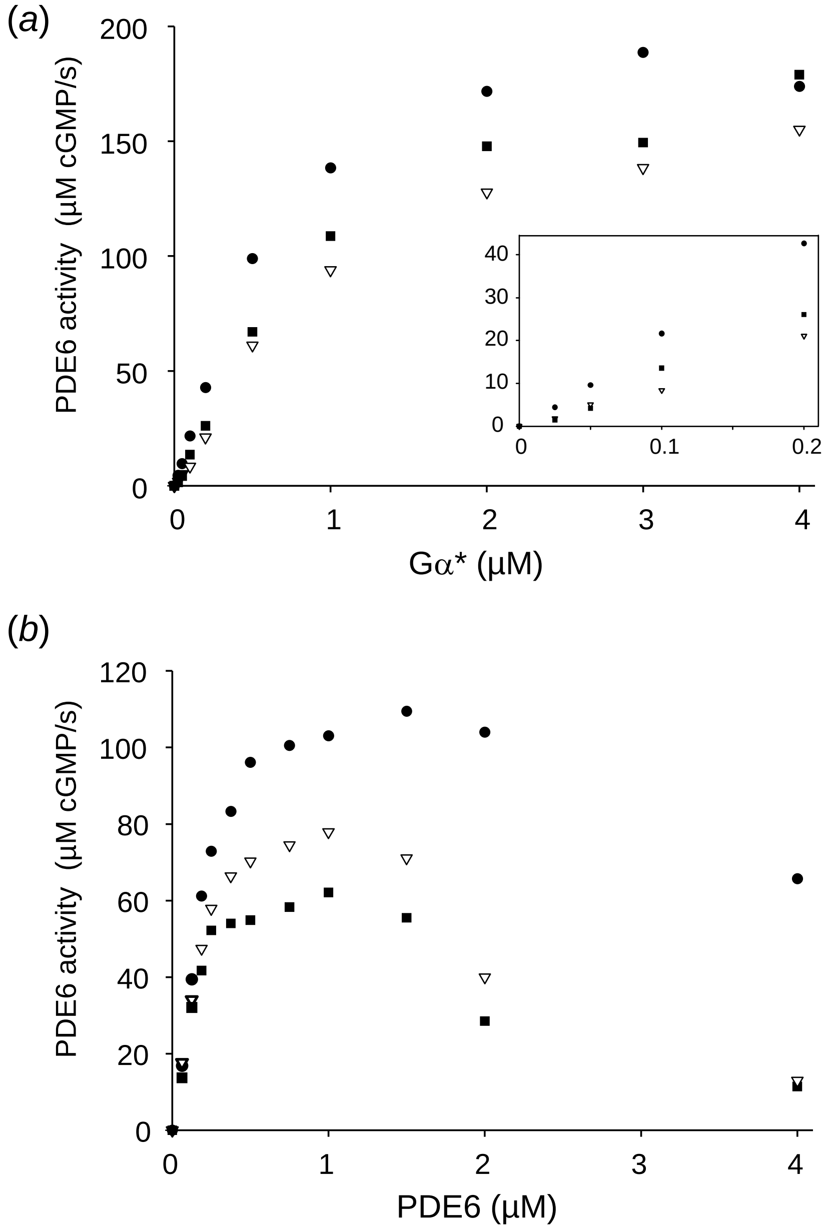


**Figure S2.** Activity of PDE6 stimulated by GTPγS-activated G protein α-subunit (Gα*). (*a*) Activity of PDE6 (0.1 µM) measured as rate of cGMP hydrolysis at increasing Gα* concentrations in the presence of disc membranes (10 µM rhodopsin; same data as in figure 1*a*) (*b*) PDE6 activity as a function of PDE6 concentration at fixed Gα* (0.25 µM) in the presence of disc membranes (10 µM rhodopsin; same data as in figure 1*c*). Different symbols identify the results of different experiments.

## S2 Appendix. Electron microscopy, image processing and rigid body docking

*Generation of the initial template structure using negative stained holo-PDE6*

For generation of an initial template structure, holo-PDE6 samples (3.5 µl, 10-20 ng/µl) were applied to freshly glow-discharged holey carbon grids (R2/4 Quantifoil grids, Quantifoil Micro Tools GmbH, Jena, Germany) covered with an additional thin carbon support film and stained using uranyl acetate (2% w/v). Transmission images were collected on a Tecnai G2 Spirit microscope (FEI, Oregon, USA) equipped with an Eagle CCD camera (FEI) at a nominal magnification of 42,000x. Images were collected at an acceleration voltage of 120 kV with a defocus range of 2.5 to 3.5 µm. The pixel-size at the object plane corresponds to 2.6 Å/pixel.

Defocus estimation was performed using CTFFIND [1]. Manual identification yielded a total of 6200 particle images from micrographs pre-selected based on calculated defocus and astigmatism. Particle images were aligned, classified by K-means and averaged in SPIDER [2]. Resulting class-averages were used to obtain template 3D structures using EMAN2 [3].

*Cryo-EM structure of tPDE6*

For the vitrified sample, a strong preferential orientation of holo-PDE6 on the carrier grid surface was observed, and we found ourselves unable to prevent this by surface modifications of the grid. Assuming that the known C-terminal lipid-modifications were responsible for this preferential orientation, we generated a lipid-free PDE6 (tPDE6) by limited trypsination of PDE6 (see Materials and methods for details). For this sample, holey carbon grids (Quantifoil) were covered with an additional thin carbon support film and glow-discharged prior to use. tPDE6 were supplemented with 1% CHAPS (3-[(3-Cholamidopropyl)dimethylammonio]-1-propanesulfonate), applied to the grid and vitrified using a Vitrobot plunger (FEI). Transmission images were collected on a Tecnai G2 Spirit

microscope (FEI) equipped with an Eagle 2k CCD camera (FEI) at 42,000x magnification using the Leginon system [4]. 1,143 micrographs were collected at an acceleration voltage of 120 kV with a defocus range of -1 to -5 µm. The pixel-size at the object plane corresponds to 2.6 Å/pixel.

Defocus estimation was performed using CTFFIND [1]. In light of the small mass of tPDE6 one has to consider the danger of simply reproducing the initial input model from images showing only noise [5]. Consequently, we only used the best 183 micrographs showing the highest image contrast, likely due to optimal ice thickness, and determined the positions of candidate particles images by hand. Thus, accidentally selected empty windows containing noise are not biased towards any template structure, as would be the case for reference-based particle identification methods. Moreover, refinement procedures were strictly limited to the low-frequency regime using low-pass filters to reduce noise-accumulation. 67,839 raw particle images were subjected to multiple rounds of multi-reference template matching and 3D K-means-like clustering [6] using the negative stain template structure as seeding volume and without enforcing symmetry. A stable subpopulation (19,716 particle images; 29 % of the dataset) was obtained and subjected to 3D variability analysis [7], showing that only distinct N- and C-terminal variable regions remained. The resulting electron density map was nearly C2 symmetric, as estimated from cross-correlation calculations, and particle images were further refined enforcing C2 symmetry. The final reconstruction reached a resolution of 28 Å according to the Fourier-shell correlation at 0.5 criteria without masking. We did not try to push the resolution beyond 28 Å, because our aim was to settle the topology of PDE6 and not to boost resolution (figures S2*f,g*).

*Three-dimensional variability analysis*

To visualize the heterogeneity of the reconstructions, three-dimensional variability [7] was calculated. In a first step, aligned projection images were grouped by their projection directions and two-dimensional variance images were calculated per group. The ensemble of variances was then backprojected in a second step to yield a three-dimensional variability volume. While this is not in a strict mathematical sense a variance, it nevertheless allows identification and visualization of regions of heterogeneity, e.g. from substoichiometric ligand binding or flexibility.

*Rigid body docking of PDE models to the electron density map of the final tPDE6 cryo-EM structure*

We compared atomic models of a nearly full-length PDE2 crystal structure [8] and a recent homology model of PDE6 [9] with our cryo-EM map of tPDE6. The GAFa/b dimer fits very well into the thinner part of our electron density map. However, using this alignment neither the catalytic domains of PDE2 (figure S3*i*) nor those of the PDE6 homology model (figures S3*j*,*k*) match the electron density of our structure. Our tPDE6 atomic model is based on the initial homology model obtained by Zeng-Elmore and colleagues [9], using the state of their atomic model prior to flexible fitting into the electron density map obtained by Goc and colleagues [10]. This model was split between the GAFa/b dimer and the catalytic domain and each part fitted individually into our cryo-EM map by sequential rigid-body fitting as implemented in Chimera [11]. Both a crossover (figure S3*l*) as well as a non-crossover arrangement of PDE6α and PDE6β chains (figure S3*m*) yields similar correlation coefficients with our map (cross-correlation coefficients of 0.893 and 0.891 for the full model). However, the crossover arrangement agrees better with inter-chain cross-linking data obtained by Zeng-Elmore and colleagues [9]. Still, there is a significant difference between the distances observed between PDE6$\alpha^{K613}$/PDE6$\beta^{K315}$ and PDE6$\alpha^{K677}$/PDE6$\beta^{K315}$. This discrepancy can be rationalized if one again assumes the catalytic domain to be flexible in the “open/active” state

of PDE6, as this would enable efficient cross-linking of both pairs of residues. Moreover, the flexibility of PDE6 C-terminal domain is also consistent with the variability in positioning of C-terminal binding prenyl binding protein or PDE6δ (PrBP/δ) with respect to the PDE6 density map [12].

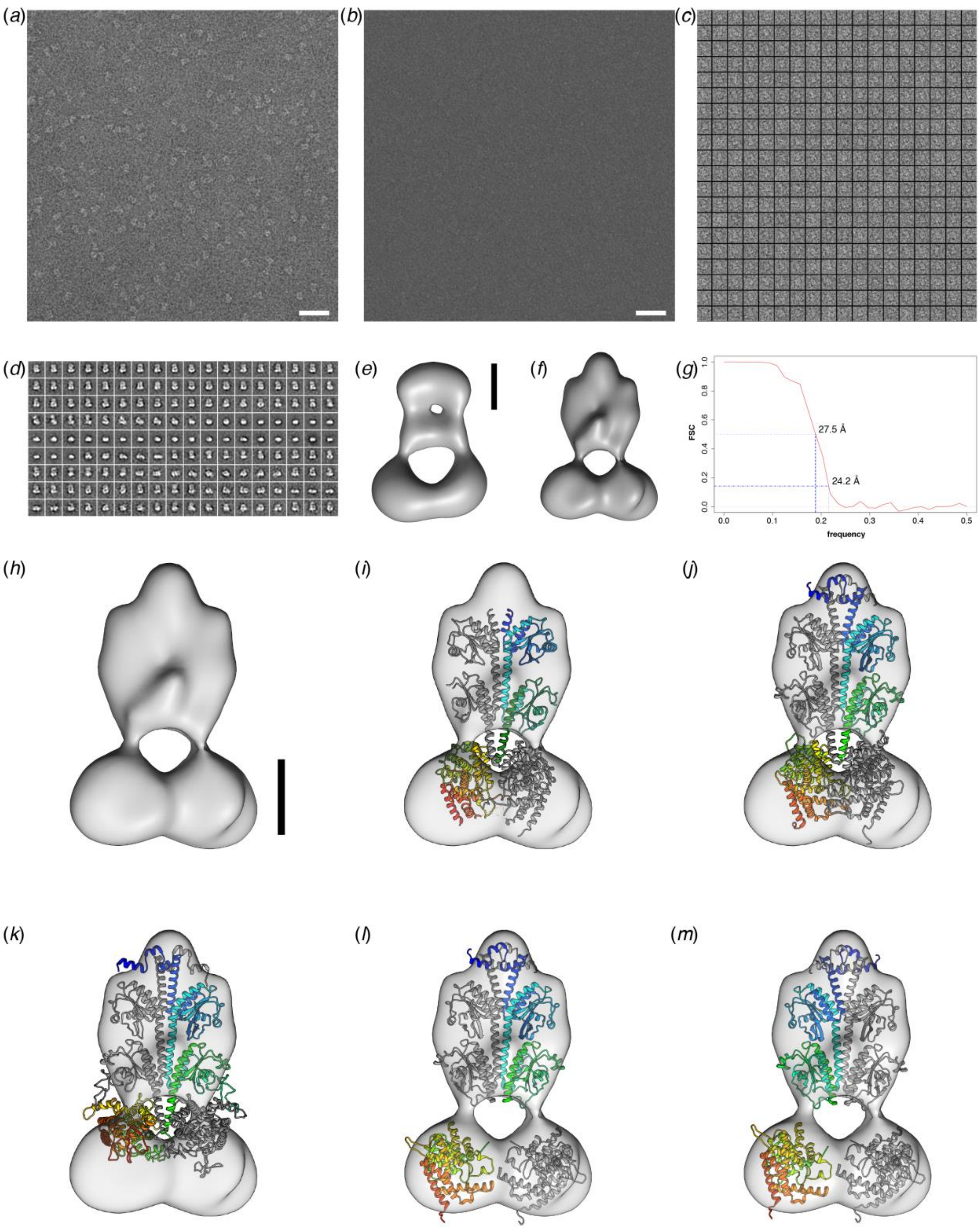


**Figure S3.** Structural analysis of PDE6. (*a*) Negative stain EM image of a representative field of holo-PDE6 (scale bar 50 nm). (*b*) Representative class averages and (*c*) corresponding initial model of negative stained holo-PDE6. (*d*) Unfiltered cryo images of tPDE6 (scale bar

50 nm). (*e*) Representative excerpt of manually selected particle images of tPDE6. Particle images have been phase-flipped to restore contrast-transfer-function distortions and filtered to approx. 20 Å for clarity. Each box has a side-length of approx. 33 nm. (*f*) Final cryo-EM map of tPDE6 shown at two different thresholds (scale bar 50 Å). (*g*) Fourier shell correlation (FSC) between even and odd particles contributing to the final tPDE6 cryo-EM structure. The resolution is in the range of 25 Å depending on the criterion used to read the resolution. (*h*) Final cryo-EM electron density map of tPDE6 (scale bar 50 Å). The following atomic models were fitted into our tPDE6 electron density map: (*i*) PDE2A crystal structure (Pandit and colleagues [8]; PDB ID 3IBJ). (*j*) Homology model of PDE6 obtained by Zeng-Elmore and colleagues [9] using an integrative modeling procedure with PDE2A serving as template. (*k*) PDE6 model obtained by Zeng-Elmore and colleagues [9] using flexible fitting of their PDE6 homology model to the electron density map of a negative stain PDE6 structure from Goc and colleagues [10]. (*l*,*m*) PDE6 model obtained by individual rigid-body docking of the Zeng-Elmore homology model [9] to the electron density map of our final tPDE6 cryo-EM structure with PDE6α and PDE6β chains in a crossover (*l*) or parallel (*m*) topology (see Materials and methods for details).

*Structural basis of the "open inhibited" state of PDE6.*

The activation of PDE2 and PDE5 [13] occurs by conformational changes resulting from cGMP binding to the GAF domains. In the X-ray structure of PDE2A in its inactive state, the enzyme displays a compact or "closed" structure. In this conformation, the two PDE2A catalytic domains mutually inhibit each other by virtue of their H-loops, each blocking substrate access to the catalytic site of the respective counterpart [8]. Pandit and colleagues [8] suggested an activation mechanism in which both catalytic subunits rotate outwards upon

cGMP binding to the GAFb domains (figure 5*a*). In this “open” conformation the catalytic sites are accessible for cGMP or cAMP. The proposed activation mechanism of PDE2A might well represent a general model of tandem GAF-containing, homodimeric PDEs such as PDE2, PDE5, PED10 and PDE11 [8]. Since the rod photoreceptor PDE6 has a similar overall domain architecture of the catalytic subunits, the question arises how its structural organization and activation mechanism compares to these PDE families. Alignment of the inactive, "closed" structure of PDE2A to the electron density of our cryo-EM structure of PDE6 reveals a significant mismatch, especially for the catalytic domains (figure S3*i*). We suggest that our cryo-EM structure of PDE6 represents the putative active, “open” conformation of PDE enzymes proposed by Pandit and colleagues [8]. The notion of an “open” conformation with flexible catalytic domains is consistent with the relative position of the domains seen in cross-linking studies of PDE6 [9]. Furthermore, the finding that the overall PDE6 structure does not change when the PDE6γ subunit is proteolyticaly removed is consistent with earlier investigations [14, 15]. Notably our active PDE6αβ structure is essentially identical to the inactive PDE6αβγγ structure obtained by Zhang and colleagues [16]. Under our experimental conditions, proteolytic removal of PDE6γ does not cause the substantial structural rearrangements observed by Zhang and colleagues [16].

## S3 Appendix. Particle-based reaction-diffusion simulations

*Simulation Setup*

Particle-based reaction-diffusion (PBRD) simulations are performed with the ReaDDy software [17]. The molecular species involved in the activation cascade R*, Gα*, PDE6, Gα*•PDE6 and Gα*•PDE6•Gα* are simulated as explicit spherical particles. Particle excluded volume is realized with repulsive harmonic potentials between the particles that is parameterized as described by Schöneberg and colleagues [18]. All particles diffuse freely on

a two-dimensional (2D) disk membrane (corresponding to the ‘free diffusion’ scenario in ref [18]. The disk has an area of 1μm$^2$, roughly corresponding to physiological parameters in mammals [19]. The membrane is realized via a harmonic membrane potential that is parameterized as in ref [18]. Initial conditions are as follows: 250 PDE6, 0 Gα*, 0 Gα*•PDE6 and 0 Gα*•PDE6•Gα* particles reside uniformly distributed on the disk membrane. Depending on the scenario, either a pool of 250 inactive G particles is uniformly placed on the membrane that randomly switch into their active form Gα* (noise scenario), or a single R* is placed randomly on the disk that subsequently creates Gα* particles.

*Particle Parametrization*

Particle radii are derived from structural information as described in ref [18]. The radius of Gα* was set to $r^{G*}$ = 1 nm (assuming that $r^{G*}$ = 1/3 $r^{G}$). The radius of R* is derived from crystal structures to have $r^{R*}$ = 2.1 nm. For PDE6 and its complexes, the same radius is assumed. Cryo-EM reports the PDE6 dimensions as 13.5 nm x 18.5 nm x 6.5 nm. Assuming that the PDE6 lies flat on the membrane, the resulting footprint corresponds to a radius $r^{PDE}$ = 8.9 nm.

The diffusion constants of R* and Gα* are derived as in ref [18], resulting in $D^{G*}$ = 1.5 μm$^2$/s and $D^{R}$ = 0.7 μm$^2$/s. Please note, that the apparent macroscopic diffusion constants are used in this study, since the crowding rhodopsin molecules are not resolved explicitly. For PDE6 and its complexes with Gα*, we again neglect the contribution of the Gα*s that are added to the complex. We use the Stokes-Einstein equation

$$D = \frac{k_B T}{6 p h r}$$

with T = 293 K (20°C), Boltzmann constant $k_B$, radius $r = r^{PDE}$ and membrane viscosity 100 cP [20], we arrive at $D_0^{PDE} = 0.24\,\mu m^2/s$. The high concentration of rhodopsin molecules ([R] = 25,000/µm$^2$) on the disk leads to severe crowding conditions. Since rhodopsin molecules are not included explicitly, this crowding has to be taken into account implicitly via the crowding factor D*([R]) = 0.5 [21], resulting in $D^{PDE} = 0.12\,\mu m^2/s$.

*Reaction Parametrization*

Particle simulations are based on the following reactions:

$$G \xrightarrow{k_0} G\alpha^*$$

$$G\alpha^* + PDE6 \underset{k_{-1}}{\overset{k_1}{\rightleftarrows}} G\alpha^* \cdot PDE6$$

$$G\alpha^* \cdot PDE6 + G\alpha^* \underset{k_{-2}}{\overset{k_2}{\rightleftarrows}} G\alpha^* \cdot PDE6 \cdot G\alpha^*$$

For the signal scenario (i.e. R* catalyzed, local Gα* production) the first rate constant $k_0$=1000/s/R* is based on the experimentally determined value [22]. For the noise scenario (i.e. spontaneous, uniform Gα* production), each of the 250 inactive G proteins is activated with a rate $k_0'$ = 1000/250/s = 4/s in order to obtain the same overall Gα* production rate as in the signal scenario.

The second and third reactions, i.e. the PDE6 complex formation reactions, involve both an on- and off-rate. Using the experimentally determined $K_d$ values ($K_{d1}$ = 19 nM, $K_{d2}$ = 586 nM), we have to fix either the $k_{on}$ or the $k_{off}$ rate to parameterize the model. We choose to set all $k_{on}$ rates to their diffusion limit and then derive $k_{off}$ via $k_{off} = K_d\, k_{on}$. The diffusion limit is given by the Smoluchowski rate $k_{on}^{diff} = 4\pi\bar{D}\bar{r}$, with $\bar{D}$ the sum of the diffusion constants of the associating molecules and $\bar{r}$ the sum of their radii. The contributions for G protein are

known from the literature $D^{G^*}$ = 1.5 μm$^2$/s [23] and $r_G$ = 1nm. Now we have all contributions to compute $k_{on}^{diff}$ : $\bar{D}$ = 1.62 μm$^2$/s , $\bar{r}$ = 9.9 nm; leading to $k_{on}^{diff}$ = 0.2 μm$^3$/s = 0.12/nM/s. Since all molecular concentrations on the disk membrane are given in 2D concentrations, we use the following formula from [22] to transfer the 3D on-rate into a 2D on-rate:

$$\frac{[X]^{3D}}{[X]^{2D}} = \frac{[R]^{3D}}{[R]^{2D}}$$

Here we assume, that $[R]^{3D}$ = 3 μM and $[R]^{2D}$ = 25,000 μm$^2$. The result is $k_{on}^{diff}$ = 1 μm$^2$/s. The same value for the G* PDE6 activation rate is supposed in ref [23].

We set $k_1 = k_2 = k_{on}^{diff}$ and arrive via the Smoluchowski equation at $k_{-1}$ = 2.4/s and $k_{-2}$ =72/s. In order to have all reaction rates in the same unit, we convert $k_0$ =1000/R*/s to $k_0$ = 1000 μm$^2$/s, assuming that we have a single R* on the disk membrane [R*] = 1 μm$^{-2}$. In order to demonstrate the validity of this parametrization, we can compare the equilibrium solution of Gα*•PDE6•Gα* using the just derived system of rates (figure S4*a*) with the experimentally measured PDE6 activity (figures 1*a* and S1*a*). The ODE model is built on this parameterization. Spatial effects, like microscopic rate constants only apply for particle based models and are derived in the following.

We now have to transform the just derived rates to the two dimensional particle model on the disk membrane. It is known, that there is a large difference in sampling efficiency of the diffusion process when it is performed in 3D or 2D [24]. In certain cases, this effect is as large as two orders of magnitude higher sampling efficiency, i.e. number of collisions between educt and product in 2D as compared as to 3D [25]. We will assume that the diffusion limited on-rate is for these reasons 10-fold higher than computed above (i.e. $k_{on}^{diff,2D}$ = 1.2/nM/s), which leads in effect to 10 fold higher off-rates for the reverse process to keep their ratios constant ($k_{-1}^{2D}$= 24/s and $k_{-2}^{2D}$ =720/s). Figure S4*b-i* shows an ODE simulation of PDE6

activation over time for an initial concentration of 30 nM PDE6, as it would be expected on a single disk membrane.

In a particle simulation like ReaDDy, second order reaction rates can no longer be used as they incorporate both a diffusion component and the actual reaction component in a single rate. The diffusion component is simulated in the simulation explicitly. Therefore, the microscopic reaction rate, i.e. the unimolecular rate, which describes that two particles react to products if they have formed an encounter complex is necessary (see ref [18] for an in depth discussion). In this case, the microscopic rate constant is parametrized by sampling of the 2D noise scenario on the disk membrane against the ODE solution. The result is $k_{on,micro}^{diff,2D}$ = 10,000/s. See figures S4*f-i* for a depiction of the resulting 2D PBRD simulation results and the ODE solution.

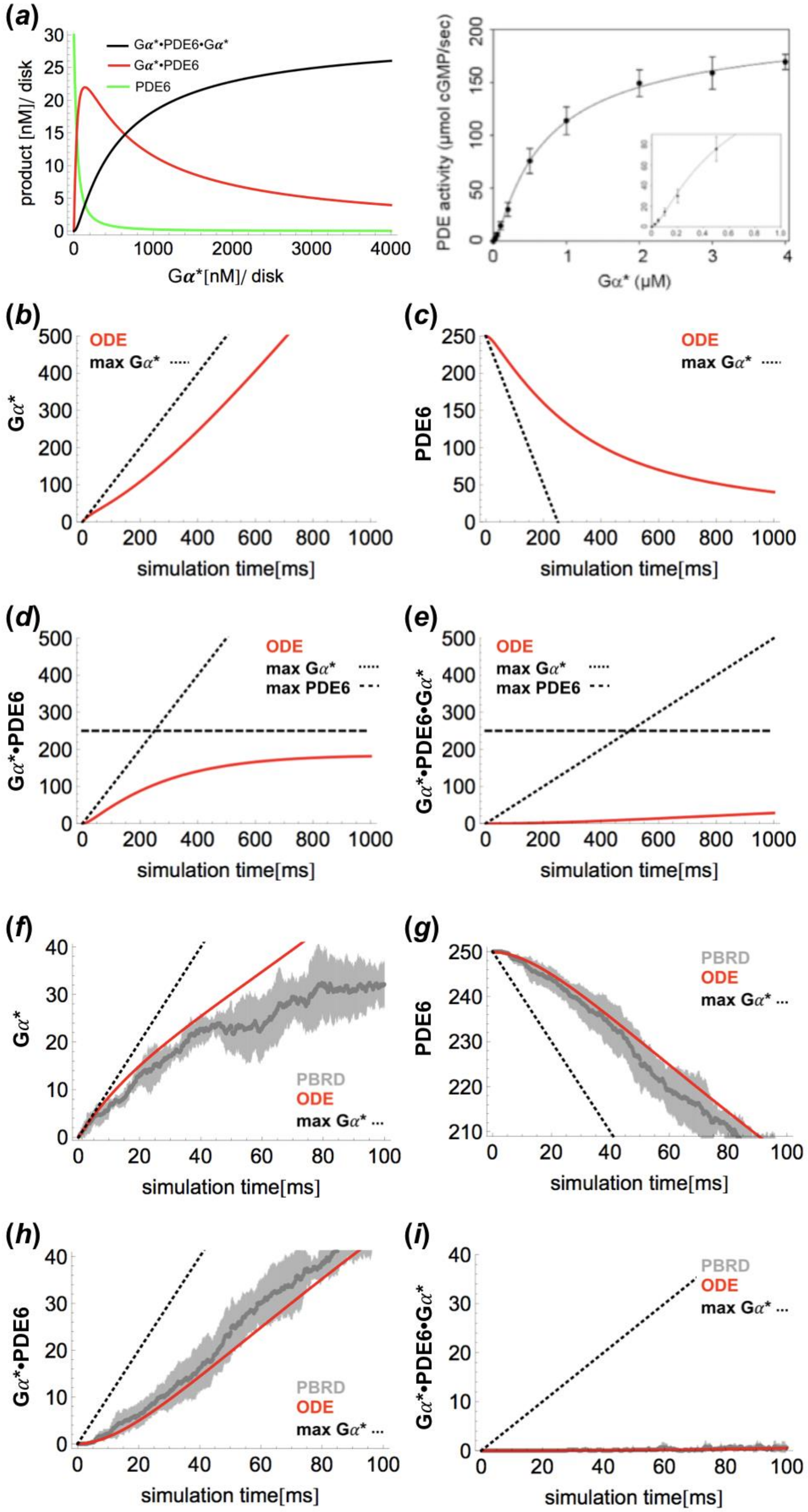

(a)
Gα*•PDE6•Gα*
Gα*•PDE6
PDE6
product [nM]/ disk
Gα*[nM]/ disk
PDE activity (μmol cGMP/sec)
Gα* (μM)
(b)
ODE
max Gα*
Gα*
simulation time[ms]
(c)
ODE
max Gα*
PDE6
simulation time[ms]
(d)
ODE
max Gα*
max PDE6
Gα*•PDE6
simulation time[ms]
(e)
ODE
max Gα*
max PDE6
Gα*•PDE6•Gα*
simulation time[ms]
(f)
PBRD
ODE
max Gα* ...
Gα*
simulation time[ms]
(g)
PBRD
ODE
max Gα* ...
PDE6
simulation time[ms]
(h)
PBRD
ODE
max Gα* ...
Gα*•PDE6
simulation time[ms]
(i)
PBRD
ODE
max Gα* ...
Gα*•PDE6•Gα*
simulation time[ms]

**Figure S4.** Comparison between ODE- and PBRD-simulations. (*a*) Plot of the ODE derived equilibrium concentration of PDE6 (green), Gα*•PDE6 (red) and Gα*•PDE6•Gα* (black), with respect to the total amount of Gα* when 30 nM PDE6 is initially present. The calculated Gα*•PDE6•Gα* production behaves similar as the measured PDE6 activity (see figure 1*a*), because >98% of the PDE6 activity is due to Gα*•PDE6•Gα*. (*b*-*e*): The ODE simulation (red curves) yields the time evolution of Gα* (*b*), PDE6 (*c*), Gα*•PDE6 (*d*) and Gα*•PDE6•Gα* (*e*) during 1s. Used are the initial condition of 30 nM PDE6 and the rates $k_{on}^{diff,2D}, k_{-1}^{2D}$ and $k_{-2}^{2D}$. The dashed lines indicate the boundaries of the system that are given by maximum Gα* that is produced over time and the maximum available PDE6 (horizontal dashed line). Note that the production rate of Gα*•PDE6•Gα* is very low, especially in the physiological relevant first hundred milliseconds. (*f*-*i*) PBRD simulation of the noise scenario (gray curves) in comparison with the ODE simulation (red curves): Gα* (*f*), PDE6 (*g*), Gα*•PDE6 (*h*) and Gα*•PDE6•Gα* (*i*). Note that the explicitly simulated diffusion in combination with $k_{on,macro}^{diff,2D}$ leads effectively to the same simulated output. The dashed lines indicate the boundaries of the system that are given by the maximum Gα* that is produced over time

**Table S3.** Parameters used in PBRD- and ODE-simulations.

| | | |
|---|---|---|
| $N_{R*,init}$ | 1 | Number of activated rhodopsin (R*) |
| $N_{G*,init}$ | 0 | Initial number of activated G protein $\alpha$-subunits (G$\alpha$*) |
| $N_{PDE6,init}$ | 250 | Initial number of PDE6 |
| $N_{G*PDE6,init}$ | 0 | Initial number of G$\alpha$*•PDE6 |
| $N_{G*PDE6G*,init}$ | 0 | Initial number of G$\alpha$*•PDE6 •G$\alpha$* |
| $r^{G*}$ | 1 nm | Reaction radius of G$\alpha$* |
| $r^{R*}$ | 2.1 nm | Reaction radius of R* |
| $r^{PDE}$ | 8.9 nm | Reaction radii of PDE6, G$\alpha$*•PDE6 and G$\alpha$*•PDE6 •G$\alpha$* |
| $D^{G*}$ | 1.5 $\mu m^2/s$ | Apparent diffusion constant of G$\alpha$* |
| $D^{R}$ | 0.7 $\mu m^2/s$ | Apparent macroscopic diffusion constant of R* |
| $D^{PDE}$ | 0.12 $\mu m^2/s$ | Apparent macroscopic diffusion constants of PDE6, G$\alpha$*•PDE6 and G$\alpha$*•PDE6 •G$\alpha$* |
| $k_0$ | 1000/s/R* | R* catalyzed G$\alpha$* production rate (signal scenario) |
| $k_0$' | 1000/s/G | Spontaneous G$\alpha$* production rate (noise scenario) |
| $k_{on}^{diff,2D}$ | 1.2/nM/s | Rate of G$\alpha$*•PDE6 and G$\alpha$*•PDE6 •G$\alpha$* formation |
| $k_{on,micro}^{diff,2D}$ | 10,000/s | Microscopic rate of G$\alpha$*•PDE6 and G$\alpha$*•PDE6 •G$\alpha$* formation |
| $k_{-1}^{2D}$ | 24/s | Rate of G$\alpha$*•PDE6 dissociation |
| $k_{-2}^{2D}$ | 720/s | Rate of G$\alpha$*•PDE6 •G$\alpha$* dissociation |
| T | 293 K (20°C) | Temperature |

**S1 Movie.** Spatio-temporal development of PDE6 activation in the PBRD signal scenario.

The video shows a PBRD simulation drawn to scale on the 1 $\mu m^2$ disk membrane, initially populated with 250 PDE6 (green), 1 R* molecule (yellow), 0 Gα* (cyan), 0 Gα*•PDE6 (red) and 0 Gα*•PDE6•Gα* (black). It is visible how Gα* diffuses from the locus of its activation by R* and binds to PDE6 molecules on its path during the 100ms simulation. The local Gα* production leads to a large local concentration and hence to efficient formation of fully active Gα*•PDE6•Gα*.

**Supplementary Information References**


1 Mindell, J. A., Grigorieff, N. 2003 Accurate determination of local defocus and specimen tilt in electron microscopy. *Journal of structural biology*. **142**, 334-347.

2 Frank, J., Radermacher, M., Penczek, P., Zhu, J., Li, Y., Ladjadj, M., Leith, A. 1996 SPIDER and WEB: processing and visualization of images in 3D electron microscopy and related fields. *Journal of structural biology*. **116**, 190-199. (10.1006/jsbi.1996.0030)

3 Tang, G., Peng, L., Baldwin, P. R., Mann, D. S., Jiang, W., Rees, I., Ludtke, S. J. 2007 EMAN2: an extensible image processing suite for electron microscopy. *Journal of structural biology*. **157**, 38-46. (10.1016/j.jsb.2006.05.009)

4 Suloway, C., Pulokas, J., Fellmann, D., Cheng, A., Guerra, F., Quispe, J., Stagg, S., Potter, C. S., Carragher, B. 2005 Automated molecular microscopy: the new Leginon system. *Journal of structural biology*. **151**, 41-60. (10.1016/j.jsb.2005.03.010)

5 Henderson, R., McMullan, G. 2013 Problems in obtaining perfect images by single-particle electron cryomicroscopy of biological structures in amorphous ice. *Microscopy*. **62**, 43-50. (10.1093/jmicro/dfs094)

6 Loerke, J., Giesebrecht, J., Spahn, C. M. 2010 Multiparticle cryo-EM of ribosomes. *Methods in enzymology*. **483**, 161-177. (10.1016/S0076-6879(10)83008-3)

7 Moriya, T., Saur, M., Stabrin, M., Merino, F., Voicu, H., Huang, Z., Penczek, P. A., Raunser, S., Gatsogiannis, C. 2017 High-resolution Single Particle Analysis from Electron Cryo-microscopy Images Using SPHIRE. *J Vis Exp*. (10.3791/55448)

8 Pandit, J., Forman, M. D., Fennell, K. F., Dillman, K. S., Menniti, F. S. 2009 Mechanism for the allosteric regulation of phosphodiesterase 2A deduced from the X-ray structure of a near full-length construct. *Proceedings of the National Academy of Sciences of the United States of America*. **106**, 18225-18230. (10.1073/pnas.0907635106)

9 Zeng-Elmore, X., Gao, X. Z., Pellarin, R., Schneidman-Duhovny, D., Zhang, X. J., Kozacka, K. A., Tang, Y., Sali, A., Chalkley, R. J., Cote, R. H., *et al.* 2014 Molecular Architecture of Photoreceptor Phosphodiesterase Elucidated by Chemical Cross-Linking and Integrative Modeling. *Journal of molecular biology*. (10.1016/j.jmb.2014.07.033)

10 Goc, A., Chami, M., Lodowski, D. T., Bosshart, P., Moiseenkova-Bell, V., Baehr, W., Engel, A., Palczewski, K. 2010 Structural characterization of the rod cGMP phosphodiesterase 6. *Journal of molecular biology*. **401**, 363-373. (10.1016/j.jmb.2010.06.044)

11 Pettersen, E. F., Goddard, T. D., Huang, C. C., Couch, G. S., Greenblatt, D. M., Meng, E. C., Ferrin, T. E. 2004 UCSF Chimera--a visualization system for exploratory research and analysis. *Journal of computational chemistry*. **25**, 1605-1612. (10.1002/jcc.20084)

12 Qureshi, B. M., Schmidt, A., Behrmann, E., Burger, J., Mielke, T., Spahn, C. M. T., Heck, M., Scheerer, P. 2018 Mechanistic insights into the role of prenyl-binding protein PrBP/delta in membrane dissociation of phosphodiesterase 6. *Nat Commun*. **9**, 90. (10.1038/s41467-017-02569-y)

13 Jäger, R., Schwede, F., Genieser, H. G., Koesling, D., Russwurm, M. 2010 Activation of PDE2 and PDE5 by specific GAF ligands: delayed activation of PDE5. *British journal of pharmacology*. **161**, 1645-1660. (10.1111/j.1476-5381.2010.00977.x)

14 Kajimura, N., Yamazaki, M., Morikawa, K., Yamazaki, A., Mayanagi, K. 2002 Three-dimensional structure of non-activated cGMP phosphodiesterase 6 and comparison of its image with those of activated forms. *Journal of structural biology*. **139**, 27-38.

15 Matte, S. L., Laue, T. M., Cote, R. H. 2012 Characterization of conformational changes and protein-protein interactions of rod photoreceptor phosphodiesterase (PDE6). *The Journal of biological chemistry*. **287**, 20111-20121. (10.1074/jbc.M112.354647)

16 Zhang, Z., He, F., Constantine, R., Baker, M. L., Baehr, W., Schmid, M. F., Wensel, T. G., Agosto, M. A. 2015 Domain Organization and Conformational Plasticity of the G Protein Effector, PDE6. *The Journal of biological chemistry*. (10.1074/jbc.M115.647636)
17 Schöneberg, J., Noe, F. 2013 ReaDDy – a software for particle-based reaction-diffusion dynamics in crowded cellular environments. *PloS one*. **8**, e74261. (10.1371/journal.pone.0074261)
18 Schöneberg, J., Heck, M., Hofmann, K. P., Noe, F. 2014 Explicit spatiotemporal simulation of receptor-G protein coupling in rod cell disk membranes. *Biophysical journal*. **107**, 1042-1053. (10.1016/j.bpj.2014.05.050)
19 Nickell, S., Park, P. S., Baumeister, W., Palczewski, K. 2007 Three-dimensional architecture of murine rod outer segments determined by cryoelectron tomography. *The Journal of cell biology*. **177**, 917-925. (10.1083/jcb.200612010)
20 Kung, C. E., Reed, J. K. 1986 Microviscosity measurements of phospholipid bilayers using fluorescent dyes that undergo torsional relaxation. *Biochemistry*. **25**, 6114-6121. (10.1021/bi00368a042)
21 Pugh, E. N., Jr., Miller, W. H. 1987 Special topic: phototransduction in vertebrates. General introduction. *Annual review of physiology*. **49**, 711-714. (10.1146/annurev.ph.49.030187.003431)
22 Heck, M., Hofmann, K. P. 2001 Maximal rate and nucleotide dependence of rhodopsin-catalyzed transducin activation: initial rate analysis based on a double displacement mechanism. *The Journal of biological chemistry*. **276**, 10000-10009. (10.1074/jbc.M009475200)
23 Pugh, E. N., Jr., Lamb, T. D. 1993 Amplification and kinetics of the activation steps in phototransduction. *Biochimica et biophysica acta*. **1141**, 111-149.
24 Berg, H. C. 1993 *Random Walks in Biology*. Princeton University Press.
25 Axelrod, D., Wang, M. D. 1994 Reduction-of-dimensionality kinetics at reaction-limited cell surface receptors. *Biophysical journal*. **66**, 588-600.
